# An observational, non-interventional study for the follow-up of patients with amyloidosis who received miridesap followed by dezamizumab in a phase 1 study

**DOI:** 10.1186/s13023-022-02405-7

**Published:** 2022-07-09

**Authors:** Duncan Richards, Helen Millns, Louise Cookson, Mary Ann Lukas

**Affiliations:** 1 University of Oxford, Oxford, UK; 2 GlaxoSmithKline, Stevenage, Hertfordshire, UK; 3 GlaxoSmithKline, Cambridge, UK; 4 GlaxoSmithKline, Philadelphia, PA USA

**Keywords:** Amyloidosis, Dezamizumab, Treatment response, Observational study, Anti-SAP treatment, Miridesap, CPHPC

## Abstract

**Background:**

Miridesap depletes circulating serum amyloid P (SAP) and dezamizumab (anti-SAP monoclonal antibody) targets SAP on amyloid deposits, triggering amyloid removal. In a phase 1, first-in-human study (FIHS), progressive amyloid removal was observed in some patients after ≤ 3 cycles of miridesap/dezamizumab.

**Methods:**

This observational, non-interventional study in patients who received miridesap/dezamizumab during the FIHS (planned follow-up: 5 years) evaluated response to treatment based on routine assessments of disease status and key organ function. In a post hoc analysis, patients responding to treatment in the FIHS during follow-up were identified as responders and further categorized as sustained or declining responders.

**Results:**

In the FIHS, 17/23 patients were treatment responders. Of these patients, seven (immunoglobulin light chain [AL], n = 6; serum amyloid A, n = 1) were considered sustained responders and ten (fibrinogen-a alpha chain [AFib], n = 5; AL, n = 4; apolipoprotein A-I, n = 1) were considered declining responders. We primarily present responder patient-level data for functional, cardiac, laboratory and imaging assessments conducted during the follow-up period, with non-responder data presented as supplementary.

**Conclusion:**

No further development of miridesap/dezamizumab is planned in amyloidosis. However, long-term follow-up of these patients may provide insight into whether active removal of amyloid deposits has an impact on disease progression.

***Trial registration*:**

ClinicalTrials.gov, NCT01777243. Registered 28 January 2013, https://clinicaltrials.gov/ct2/show/study/NCT01777243.

**Supplementary Information:**

The online version contains supplementary material available at 10.1186/s13023-022-02405-7.

## Introduction

The amyloidoses are a group of rare and often fatal diseases in which misfolded proteins form insoluble amyloid fibrils that accumulate in vital organs, such as the heart, kidneys and liver, causing progressive dysfunction [1, 2]. Tissue and system involvement vary by amyloidosis subtype, and the disease can be acquired or hereditary. Of 36 proteins identified in humans that may form amyloid fibrils, around half have been associated with systemic amyloidosis, while the remainder are associated with localized disease [1]. The most common amyloidosis subtypes, immunoglobulin light chain (AL), serum amyloid A (AA), and transthyretin (ATTR), are associated with systemic disease [1, 3]. Other proteins associated with systemic disease include fibrinogen-a alpha chain (AFib) and apolipoprotein A-I (ApoAI) [1]. Survival estimates vary by subtype and organ involvement, but cardiac involvement is the most important determinant of clinical outcomes; patients with cardiac involvement have lower survival rates than those without [4, 5]. In patients with AL amyloidosis, median survival has improved over time, from 0.77 years during 1995–1999 to 3.5 years during 2010–2013. However, in patients with Mayo stage II or III cardiac involvement, only marginal or no improvement in survival has been observed over time from 2000 to 2014 [6].

Amyloidosis is difficult to diagnose; in a survey of 533 patients with amyloidosis, 37.1% reported that they did not receive an amyloidosis diagnosis until ≥ 1 year after their initial symptoms [7]. Once diagnosed, management of amyloidosis involves support for damaged organs, combined with reduction of amyloid protein production when possible, for example, by chemotherapy for AL amyloidosis [2]. Several novel therapies for ATTR have been approved in recent years: a small molecule TTR stabilizer (tafamidis [8]) and two nucleotide therapies (inotersen [9] and patisiran [10]). In hereditary forms of systemic amyloidosis, such as AFib and AApoA1, the production of amyloid protein is continuous, with no current therapies suppressing this process. Therefore, any effect of treatments that remove existing amyloid deposits is transient as amyloid deposition continues post treatment.

Serum amyloid P component (SAP) is a plasma protein that is universally present on amyloid deposits [11], making it a possible therapeutic target for all forms of systemic amyloidosis. Short-term administration of the small-molecule drug miridesap [(R)-1-[6-[(R)-2-carboxy-pyrrolidin-1-yl]-6-oxo-hexanoyl]pyrrolidine-2-carboxylic acid (CPHPC)] depletes circulating SAP [12, 13], but some SAP remains in amyloid deposits [14]. Dezamizumab is a fully humanized anti-SAP monoclonal antibody, which targets SAP on amyloid deposits, and triggers removal of amyloid through a macrophage giant cell response [13, 15].

In a phase 1, first-in-human study (FIHS) (NCT01777243; GSK study identifier SAP115570) the efficacy and safety of up to 3 cycles of treatment with miridesap and dezamizumab were assessed in patients with AL, AA, ATTR, AFib and ApoAI amyloidosis [16, 17]. Progressive removal of amyloid in the liver, spleen and/or kidney was observed in some patients, consistent with active removal of amyloid deposits from these organs by treatment with miridesap/dezamizumab. In terms of safety, there were no observed direct adverse effects on organ function, but infusion-related effects were observed in the majority of patients receiving effective doses [16].

Although no further development of dezamizumab anti-SAP treatment in amyloidosis is planned, long-term follow-up of patients from the FIHS may provide insight into whether active removal of amyloid deposits has an impact on the progression of disease. This was an observational, non-interventional post hoc study of patients after receiving miridesap/dezamizumab during the phase 1 FIHS. In this report we focus on characterization of patients according to their response to treatment in the FIHS and whether this response was maintained during follow-up.

## Methods

### Study design and methods

#### Phase 1 parent study

**Fig. 1:**
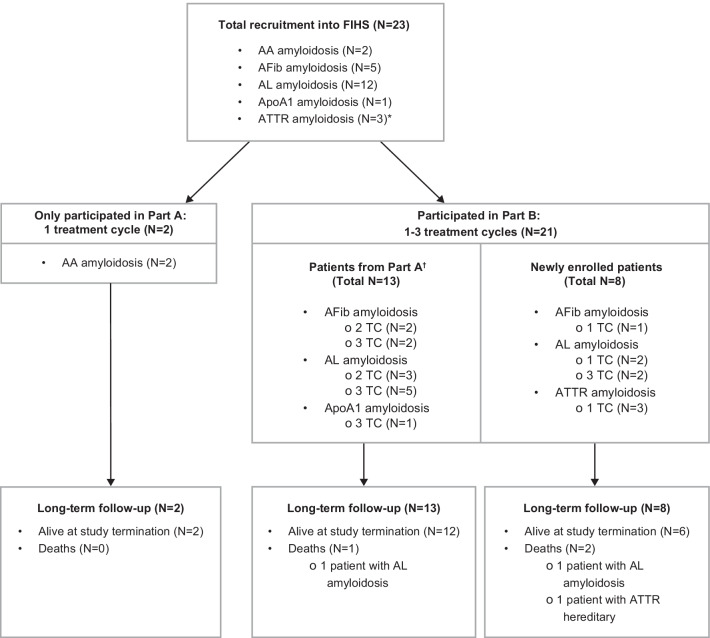
Study flow diagram. *Includes two patients with wild-type ATTR; one patient with hereditary ATTR. AA, serum amyloid A; ApoAI, apolipoprotein A-I; AFib; fibrinogen-a alpha chain; AL, immunoglobulin light chain; ATTR, transthyretin

The open-label, single-dose-escalation, non-randomized phase 1 trial [16, 17] enrolled and treated a total of 23 patients, aged 44–69 years (inclusion criterion 18–70 years), with comprehensively characterized and biopsy-proven systemic amyloidosis (AL, AA, ATTR, AFib and ApoAI). This was a two-part study; full details of the study design have been published previously and are summarized in Fig. 1 [16, 17].

Briefly, eligible patients were under the care of the UK National Health Service National Amyloidosis Centre at the Royal Free Campus of University College London, gave written informed consent, had adequate venous access, met strict functional status and organ function criteria, and were able to tolerate the study protocol. In Part A, the first six patients chosen for treatment had small or moderate amyloid loads, as determined by ^123^I-SAP scintigraphy [18, 19], eight of the nine subsequent patients selected for assessment had substantial hepatic involvement, as hepatic amyloid can be quantified with multiple independent methods. For safety reasons, patients with evidence of cardiac involvement were excluded from Part A of the study. However, after establishing acceptable safety and tolerability and following a protocol amendment, patients with cardiac involvement were included in Part B, provided cardiac function criteria were met [16]. Six patients with mild but definite cardiac involvement, three with AL and three with ATTR amyloidosis subtypes, were enrolled for preliminary safety assessment.

In Part A [17], 15 patients received miridesap intravenously (IV) over ~ 3 days to deplete circulating SAP to < 2.0 mg/L, followed by dezamizumab IV. The dezamizumab dose was 5 mg for the first two patients and was escalated in a stepwise fashion (and/or adjusted based on amyloid load) up to a maximum of 2000 mg. Miridesap treatment continued after dezamizumab infusion to maintain SAP depletion (typically for 11 days). Thirteen of the fifteen subjects from Part A and eight newly enrolled subjects were included in Part B [16] to investigate the safety, tolerability and efficacy of up to three cycles of anti-SAP treatment. All patients received one to three treatment cycles and there was no control group.

#### Non-interventional follow-up study

The present observational post hoc study (NCT01777243) was initiated on 27 May 2015 and terminated on 1 October 2018. This was an early termination following sponsor decision not to proceed with further development of miridesap/dezamizumab in amyloidosis. Planned duration was up to 5 years after the last patient had received their final dose in the FIHS. The present study took place at the UK National Amyloidosis Centre. Patients who had received miridesap followed by dezamizumab and completed the follow-up in the phase 1 trial were invited to participate. Participants underwent their usual clinical visits, and relevant data related to overall clinical status and key organ function were collated from the UK National Amyloidosis Centre database from diagnosis and included a baseline (defined as the date of dezamizumab administration in the FIHS) and subsequent visits for up to 5 years post last dose in the FIHS. Where possible, the same information was collated from diagnosis until entry into this follow-up study. Patients underwent usual standard of care during follow-up and did not undertake any additional visits or investigations as part of this study; therefore, the interval between clinic visits varied considerably, ranging from a few days between visits to approximately 1.5 years. The full study protocol is provided as Additional file 1.

### Ethics

The study protocol, any amendments, the informed consent, and other information that required pre-approval were reviewed and approved by a single investigational ethics committee (Wales Research Ethics Committee, Cardiff, UK), in accordance with the International Conference on Harmonisation of Technical Requirements for Registration of Pharmaceuticals for Human Use Good Clinical Practice and applicable country-specific requirements. Written informed consent was obtained from each patient.

The datasets used during the current study are available from www.clinicalstudydatarequest.com on reasonable request.

### Outcome measures

Endpoints included parameters which were collected as part of routine standard of care for patients with amyloidosis, such as assessments of disease type, survival outcome, functional status, biomarkers of key organ function and disease status. As specified in the study protocol (Additional file 1), endpoints varied according to the subtype of amyloidosis. Functional assessments included the 6-min walking distance (6MWD), New York Heart Association (NYHA) Class, and Eastern Cooperative Oncology Group (ECOG) performance status. Cardiac assessments included levels of the cardiac biomarker N-terminal-pro B-type natriuretic peptide (NT-proBNP) and echocardiogram structural (left ventricle [LV] septum, LV posterior wall thickness) and functional parameters (early mitral inflow velocity and mitral annular early diastolic velocity [E:E′] ratio, left ventricular ejection fraction [LVEF]). Laboratory assessments of liver and renal function were measurements of gamma-glutamyl transpeptidase (GGT) and estimated glomerular filtration rate (eGFR) levels, respectively. Assessments of disease status included imaging results (^123^I-SAP scintigraphy scans [except patients with ATTR] or 3,3-diphosphono-1,2-propanodicarboxylic acid [DPD] scans [patients with ATTR only]), Mayo disease stage (AL only), free light chain (FLC) data (AL only) and serum amyloid A levels (AA only). SAP scan results were used to assess overall and organ-specific (e.g. liver, spleen, kidney and adrenals) amyloid load over time. Overall amyloid load was categorized by a single expert reader according to the uptake of ^123^I-SAP in the organs and the signal of the residual blood-pool 24 h after tracer injection. The overall amyloid load was categorized as none, small (definite organ uptake but substantial blood-pool signal), moderate (more intense organ localization and reduced blood-pool signal) or large (very strong organ localization with little or no blood-pool activity), and the organ-specific amyloid load as normal or abnormal. It was also noted whether the amyloid load was better, stable or worse compared with the previous visit.

### Response classification

In this analysis, response status during the FIHS and during follow-up was based on post hoc clinical review of the available data. Due to the exploratory nature of the study and the small number of patients involved with each amyloidosis subtype, it was not possible to apply a standardized classification. Decisions regarding response status were guided by the criteria listed in Table 1; these criteria were not pre-defined prior to the study. Responses were assessed by a clinical scientist and reviewed by a study physician; revision of response classification was reviewed by collective discussion. Response classification accounted for the organ site(s) involved; in patients with kidney involvement, for example, changes in eGFR were given particular focus in assigning a response status. No data were concealed at the time of making this assessment. Where there was narrative record of clonal relapse (patients with AL) the involved FLC or kappa/lambda ratio were reviewed for potential concomitant changes; these were not used to determine response status.

**Table 1:** Criteria used to guide response classification in this analysis

Criterion	Response during FIHS	Response during follow-up	Decline during follow-up
Amyloid load	Improvement* in SAP scan from baseline^†^ (pre-dose) at any study visit	Stable or improvement* in SAP scan compared with amyloid load from FIHS	Worsening in SAP scan compared with amyloid load from FIHS
6MWD	Not applicable	Stable or an increase ≥ 30 m [27] compared with the maximum 6MWD observed during the FIHS	Decrease ≥ 30 m compared with the maximum 6MWD observed during the FIHS
GGT (only in patients with abnormal GGT and/or hepatic amyloid during the FIHS)	Decrease (> 25%) in GGT from baseline^†^ (pre-dose) at any study visit	Stable or decrease (of any magnitude) compared with the final GGT level observed during the FIHS	Increase (of any magnitude) compared with the final GGT level observed during the FIHS
eGFR	No formal measure of response. Absolute values reviewed for safety and post-treatment response	Met criteria for an improvement in CTCAE grade [28] in kidney function at any point during follow-up	Met criteria for a decline in CTCAE grade in kidney function at any point during follow-up
NT-proBNP	No formal measure of response. Reviewed in respect of post-dose transient response in cardiac amyloid patients and in relation to eGFR in renal amyloid patients	Stable or decrease (> 30% reduction and > 300 ng/L [29]) compared with the last NT-proBNP level observed during the FIHS	Increase (> 30% and > 300 ng/L) compared with the last NT-proBNP level observed during the FIHS

*Improvement was based on subjective assessment of patient data

^†^The baseline date was defined as the date of first pharmacologically active administration of dezamizumab (i.e. ≥ 200 mg) in the FIHS. For the one subject who received a non-pharmacologically active dose (i.e. < 200 mg), their baseline date was defined as the date of first administration of a non-pharmacologically active dose of dezamizumab

6MWD, 6-min walking distance; CTCAE, Common Terminology Criteria for Adverse Events; eGFR, estimated glomerular filtration rate; FIHS, first-in-human study; GGT, gamma-glutamyl transpeptidase; NT-proBNP, N-terminal-pro B-type natriuretic peptide; SAP, serum amyloid P

Response status during the FIHS was defined as a meeting one or more of the criteria (Table 1) at one or more visits during the study. After review of the follow-up data, patients were classified as a ‘sustained responder’ (showing a response in the FIHS, maintained over the follow-up period); a ‘declining responder’ (showing a response in the FIHS, not maintained over the follow-up period); or a ‘non-responder’ (showing no response in the FIHS or during the follow-up period). Responder classification was assigned based on maintained long-term trends irrespective of transient changes such as those associated with a clonal relapse that was subsequently treated. There were no defined guidelines relating to how many visits an improvement/decline should be observed before making a decision on response classification; similarly, there were no guidelines relating to the number of response criteria that had to be met, or their relative importance. The final response classification was based on the authors’ interpretation of all available follow-up data and knowledge of each patient’s response to treatment in the FIHS. As such, a patient meeting a criterion for response during follow-up could be defined as a ‘declining responder’ based on evidence from other response criteria.

### Statistical analysis

Analyses were performed after the database was locked following study termination. The baseline date was defined as the date of first pharmacologically active administration of dezamizumab (i.e. ≥ 200 mg) in the FIHS. For the one subject who only received a non-pharmacologically active dose (i.e. < 200 mg), their baseline date was defined as the date of first administration of a non-pharmacologically active dose of dezamizumab.

This study was not designed to test for a difference between the phase 1 study dosing groups or disease types. All analyses are considered exploratory, and no formal hypotheses were tested. Point estimates and corresponding 95% confidence intervals (CI) were constructed for survival analyses where appropriate.

A Kaplan–Meier plot of survival proportion with 95% CI against time from baseline was produced together with a summary of percentiles of survival time from baseline. For subjects surviving to the end of the study, survival time was calculated as the time to the date of study termination. Date of death was recorded for any deaths during the study and survival time was calculated as time to death. Survival times were relative to the date of baseline.

## Results

### Demographics and classification

This follow-up study included all 23 patients from the FIHS; patient demographics and dosing information are provided in Table 2. The most common amyloid type was AL (n = 12). All patients were Caucasian. Patients were categorized based on hepatic organ involvement, renal involvement or cardiac involvement: hepatic only n = 8 (AL, n = 7; ApoA1 n = 1); hepatic and renal (AL, n = 2); renal only n = 7 (AFib, n = 5; AA, n = 2); cardiac only n = 4 (AL, n = 1; ATTR n = 3 [1 hereditary; 2 wild-type]); and cardiac and renal (AL, n = 2). Other organs may also have been affected, such as the spleen, bone marrow and adrenals. Median follow-up time from baseline to study termination or death was 46 months. The range of follow-up time from baseline to study termination was 34.7–63.9 months. Pre-baseline functional characteristics, which capture the latest value in the database prior to dosing, are included in Additional files 3, 4 and 5 for ECOG performance status, NYHA Class and 6MWD.

**Table 2:** Patient demographics and exposure

Patient number	Amyloid type	Site(s) of involvement*	Age^†^ (years)	Gender	Time from diagnosis to baseline^‡^ (months)	Time from baseline to study termination date or death (months)	Assessments during follow-up study, n	Doses received	Time from final dose to final follow-up (months)
001	AA	Kidney	60	Female	27	63.9	6	1	63.9
012	AA	Kidney	62	Female	27	54.7	4	1	54.7
102	AFib	Kidney	58	Male	53	43.9	7	2	43.9
104	AFib	Kidney	68	Male	55	46.0	16	3	40.7
105	AFib	Kidney	60	Male	38	47.1	12	3	43.7
106	AFib	Kidney	62	Female	158	43.7	3	2	43.7
107	AL	Liver	65	Female	126	46.9	5	3	41.4
108	AL	Liver and kidney	61	Male	12	58.0	9	2	47.1
109	ApoA1	Liver	46	Female	52	56.3	12	3	41.6
110	AL	Liver	60	Male	24	55.9	8	3	43.4
111	AL	Liver	63	Female	90	30.2^§^	5	3	18.9
113	AL	Liver and kidney	49	Female	72	51.7	4	2	46.0
114	AL	Liver	53	Male	21	51.3	8	3	42.7
115	AL	Liver	67	Female	29	50.4	6	2	38.4
116	AL	Liver	44	Female	19	49.9	8	3	41.1
117	AFib	Kidney	69	Male	6	42.9	11	1	42.9
118	AL	Cardiac	50	Male	48	41.6	9	3	36.3
119	AL	Liver	69	Female	53	41.6	5	1	41.6
120	AL	Cardiac and kidney	50	Male	86	10.8^§^	2	1	10.8
121	AL	Cardiac and kidney	47	Female	23	41.2	6	3	35.8
123	ATTR (hereditary)	Cardiac	66	Male	18	33.5^§^	6	1	33.5
124	ATTR (wild-type)	Cardiac	68	Male	18	35.0	6	1	34.9
125	ATTR (wild-type)	Cardiac	66	Male	5	34.7	7	1	34.7

*Other organs may also have been affected, for example, spleen, bone marrow, adrenals

^†^At entry into FIHS

^‡^The baseline date was defined as the date of first pharmacologically active administration of dezamizumab (i.e. ≥ 200 mg) in the FIHS. For the one subject who received a non-pharmacologically active dose (i.e. < 200 mg), their baseline date was defined as the date of first administration of a non-pharmacologically active dose of dezamizumab

^§^Patient died

AA, serum amyloid A; AFib, fibrinogen A alpha-chain; AL, immunoglobulin light chain; ApoA1, apolipoprotein A-I; ATTR, transthyretin

Of the 23 patients included in this analysis, seven patients were classified as sustained responders (hepatic, n = 3; hepatic and renal, n = 2; renal, n = 1; cardiac and renal, n = 1) and ten were classified as declining responders (hepatic, n = 4; renal, n = 5; cardiac, n = 1). Five patients were classified as non-responders. Four of these patients had had cardiac involvement; the fifth patient had hepatic involvement. One patient received a non-therapeutic dose of dezamizumab in the FIHS and was not included in the response classification. Table 3 summarizes the evidence supporting the classification of response status during the FIHS and during follow-up.

**Table 3:** Classification of patients in this analysis

Patient	Amyloid type	Classification	Basis of classification		
			Evidence for response in parent study	Evidence for sustained response	Evidence for declining/lack of response
ORGAN: LIVER					
107	AL	Sustained responder	↓ Total amyloid load on SAP scan ↑ eGFR (small)	↓ GGT Stable eGFR Stable total amyloid load on SAP scan ↑ 6MWD Stable FLC κ:λ	–
109	ApoA1	Declining responder	↓ Total amyloid load on SAP scan ↓ GGT		Worsening amyloid load on SAP scan ↑ GGT after 1 year ↑ Total amyloid load after 1 year ↓ eGFR after 1 year
110*	AL	Sustained responder	↓ hepatic amyloid load on SAP scan ↓ GGT	Stable total amyloid load on SAP scan Stable GGT	
111^†^	AL	Declining responder	↓ hepatic amyloid load on SAP scan (1st treatment)		↓ eGFR after 1 year (with associated ↑ NT-proBNP) ↑ GGT after 1 year Death
114^‡^	AL	Declining responder	↓ hepatic amyloid load on SAP scan ↓ GGT	Fluctuating amyloid load on SAP scan (worsening/better) Stable GGT	Variable total amyloid load ↓ eGFR after 2 years (with associated small ↑ NT-proBNP)
115	AL	Declining responder	↓ splenic amyloid load on SAP scan	Stable or better amyloid load on SAP scan Stable GGT	↓ eGFR (with associated small ↑ NT-proBNP) ↓ 6MWD
116	AL	Sustained responder	↓ splenic and hepatic amyloid load on SAP scan ↓ GGT	↓ GGT ↓ Total amyloid load on SAP scan (transient) ↑ 6MWD (transient)	–
119	AL	Non-responder	–	Stable eGFR Stable GGT ↑ 6MWD (slight)	Variable FLC κ:λ
ORGAN: LIVER AND KIDNEY					
108	AL	Sustained responder	↓ Total amyloid load on SAP scan ↓ GGT	Stable total amyloid load on SAP scan ↓ GGT ↑ 6MWD Stable eGFR Stable FLC κ:λ	–
113^§^	AL	Sustained responder	↓ Total amyloid load on SAP scan	↓ Total amyloid load on SAP scan Stable eGFR	
ORGAN: KIDNEY					
001	AA	N/A (non-therapeutic dose in FIHS)	N/A	N/A	N/A
012	AA	Sustained responder	↓ Renal amyloid load on SAP scan Stable eGFR	Stable total amyloid load on SAP scan Stable eGFR, stable 6MWD	–
102	AFib	Declining responder	↓ Total amyloid load on SAP scan	Stable total amyloid load on SAP scan	↓ eGFR to end stage renal disease after 2 years; double renal transplant after 3 years (with associated ↑ NT-proBNP)
104	AFib	Declining responder	↓ Splenic and renal amyloid load on SAP scan		↑ Amyloid load after ~ 3 years ↓ eGFR after 2 years (with associated ↑ NT-proBNP)
105	AFib	Declining responder	↓ Renal amyloid load on SAP scan		↓ eGFR
106	AFib	Declining responder	↓ Renal amyloid load on SAP scan	Stable total amyloid load on SAP scan Stable eGFR	↓ 6MWD
117	AFib	Declining responder (declined at 3 year)	↓ Total amyloid load on SAP scan	Stable total amyloid load on SAP scan	↓ eGFR (dialysis considered)
ORGAN: CARDIAC					
118^¶^	AL	Declining responder	↓ Splenic amyloid load on SAP scan (none detected after treatment)		Reoccurrence of small amyloid load on SAP scan ↑ NT-proBNP after 2 years ↑ GGT after 2 years ↓ 6MWD Variable FLC κ:λ
123	ATTR (hereditary)	Non-responder	–	–	↑ NT-proBNP Death
124	ATTR (wild-type)	Non-responder	–	–	↑ NT-proBNP
125	ATTR (wild-type)	Non-responder	–	–	↑ NT-proBNP with AF
ORGAN: CARDIAC AND KIDNEY					
120	AL	Non-responder	–	–	↑ GGT (slight) ↑ NT-proBNP at 7 months ↓ eGFR at 7 months Death
121	AL	Sustained responder	↓ LV mass on MRI scan ↓ splenic amyloid load on SAP scan (none detected after treatment)	↓ NT-proBNP stable (outside normal range)	

Detailed information on response classification is given in the methods section. Decisions regarding response status were guided by the criteria listed in Table 1. Responses were classified by a clinical scientist and reviewed by a study physician; response classification accounted for the organ site(s) involved. Sustained responders showed a response in the FIHS, maintained over the follow-up period; declining responders showed a response in the FIHS, not maintained over the follow-up period; non-responders showed no response in the FIHS or during the follow-up period

*Patient 110 experienced clonal collapse during the FIHS, ~ 2 months after first treatment session. During follow-up FLC κ:λ started to fall ~ 31 months after the last treatment session, indicating relapse; treatment was received

^†^Patient 111 experienced clonal relapse in the FIHS after first treatment session in study; ~ 1 year after the study, GGT and NT-proBNP significantly increased with a decline in eGFR, at which time the patient received treatment for clonal relapse

^‡^Patient 114 experienced clonal relapse during the FIHS and during the follow-up period; their amyloid load varied due to these relapses

^§^During the FIHS, patient 113 had clonal relapse between treatment sessions as evidenced by high FLC κ:λ ratio; the patient underwent treatment and FLC κ:λ improved

^¶^Patient 118 experienced clonal relapse during follow-up (increase in FLC and fall in FLC κ:λ ratio); treatment was administered

6MWD, 6-min walking distance; AA, serum amyloid A; AF, atrial fibrillation; AFib, fibrinogen A alpha-chain; AL, immunoglobulin light chain; ApoA1, apolipoprotein A-I; ATTR, transthyretin; eGFR, estimated glomerular filtration rate; FLC, free light chain; GGT, gamma-glutamyl transferase; NT-proBNP, N-terminal-pro B-type natriuretic peptide

Initial Mayo stage assessment at 1.1–3.1 months post diagnosis was available for 8/12 patients with AL; the assessments were performed ~ 1–4 years prior to entry into FIHS. In sustained responders (n = 4), three patients (108, 110 and 116, all with hepatic involvement) were Mayo stage I and one (121, cardiac involvement) was Mayo stage II. In declining responders with an initial Mayo stage assessment, two patients with hepatic involvement (114 and 115) were stage I and one patient with cardiac involvement (118) was stage IIIA. Patients 114 (clonal relapse) and 118 showed decline approximately 2 years after treatment and patient 115 showed a gradual decline throughout the follow-up period. One non-responder (119) was Mayo stage I and had hepatic involvement.

Patient outcomes during follow-up for sustained and declining responders are described below. Survival outcomes are described for all patients (sustained/declining responders and non-responders). Description of other outcomes for non-responders can be found in Additional file 2.

### Survival

**Fig. 2:**
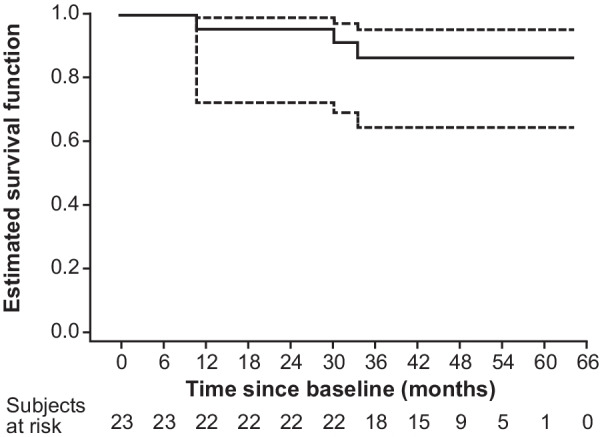
Kaplan–Meier plot of survival proportion with 95% confidence interval according to time from baseline. Dashed line, 95% confidence interval; solid line, Kaplan–Meier survival curve

Three patients (AL, n = 2 [patients 111 and 120]; hereditary ATTR, n = 1 [patient 123]) died during follow-up. Their ages at entry into the FIHS were 63, 50 and 66 years, time from diagnosis to death was 121.0, 96.5 and 51.6 months, and time from baseline to death was 30.2, 10.8 and 33.5 months, respectively. Two of these patients were classified as non-responders (120 and 123, both with cardiac involvement), and one a declining responder (111, hepatic involvement). Patient 111 had clonal relapse and accumulation of amyloid between treatment sessions in the FIHS. Overall, 100% of sustained responders (7/7 patients), 90% of declining responders (9/10 patients), and 60% of non-responders (3/5 patients) were alive at the end of the follow-up period/study termination. The Kaplan–Meier plot for survival is shown in Fig. 2.

### Functional assessments (6MWD, ECOG performance status and NYHA class)

**Fig. 3:**
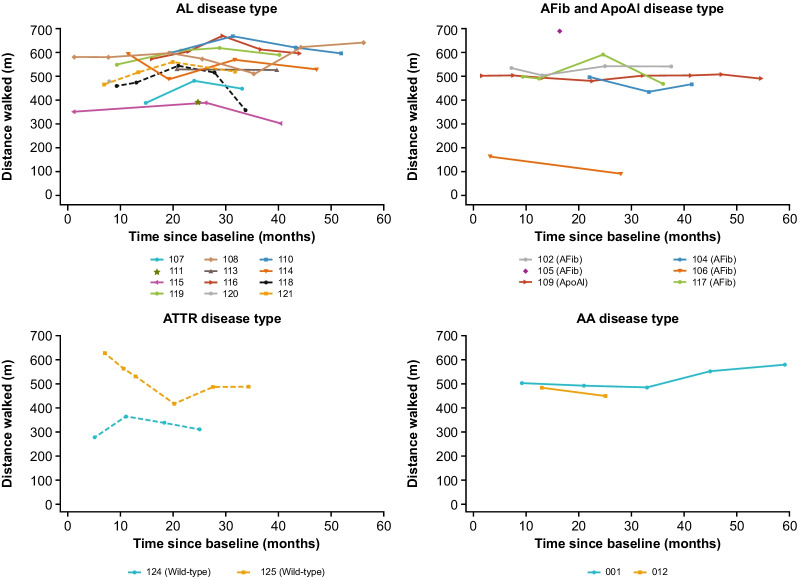
6MWD over time by patient. Dashed line, cardiac involvement; solid line, no cardiac involvement. 6MWD, 6-min walking distance; AA, serum amyloid A; ApoAI, apolipoprotein A-I; AFib, fibrinogen-a alpha chain; AL, immunoglobulin light chain; ATTR, transthyretin

The 6MWD over time is shown by amyloidosis subtype in Fig. 3. ECOG performance status, NYHA Class and 6MWD at each visit are summarized by patient in Additional files 3, 4 and 5, respectively.

#### Sustained responders

Four of the seven patients who were classed as sustained responders (107, 108, 116 and 121) showed an improvement of ≥ 30 m in 6MWD during the follow-up period. All four of these patients had AL amyloidosis. In the other three sustained responders, 6MWD remained stable (110 [AL], 113 [AL], 012 [AA]) over follow-up.

ECOG performance status was mostly 0 or 1 for all sustained responders throughout follow-up, except one assessment for patient 110 (AL), who had a score of 2 at their last follow-up visit. NYHA Class was I or II for all sustained responders throughout follow-up, except patient 121 (AL) who reached NYHA Class IV at their final follow-up visit.

#### Declining responders

6MWD remained stable in six of the ten declining responders, fluctuated for one patient (117 [AFib]) but decreased during follow-up period in patients 106 (AFib) and 115 and 118 (both AL).

ECOG performance status was mostly 0 or 1 for all declining responders throughout follow-up, except patient 104 (AFib) who had a score of 2 at their final follow-up visit, and patient 118 (AL), whose ECOG status fluctuated between 0 and 2. NYHA Class was I or II for all declining responders throughout follow-up, except patient 118 (AL) who was Class III at their penultimate follow-up visit but returned to Class II at their final visit.

### GGT assessments in patients with hepatic involvement

**Fig. 4:**
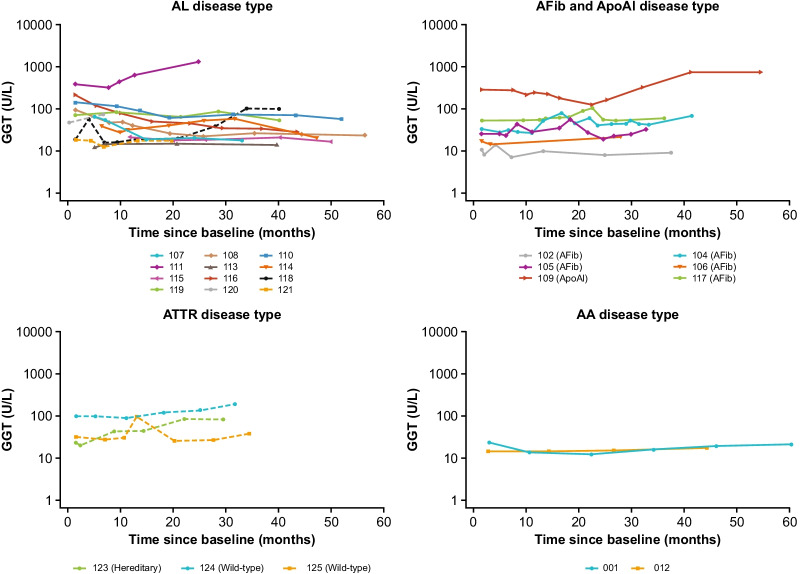
GGT over time by patient (semi-log). Dashed line, cardiac involvement; solid line, no cardiac involvement. AA, serum amyloid A; ApoAI, polipoprotein A-I; AFib, fibrinogen-a alpha chain; AL, immunoglobulin light chain; ATTR, transthyretin; GGT, gamma-glutamyl transferase

Patient-level assessments (GGT) are summarized by disease type in Fig. 4. Ten patients with hepatic involvement were enrolled in the FIHS. Of these patients, five were defined as sustained responders and four as declining responders. The remaining patient was a non-responder (Additional file 2).

#### Sustained responders

The five sustained responders with hepatic involvement had AL amyloidosis (patients 107, 108, 110, 113, 116). The improved liver function observed in patients 108 and 116 during the FIHS continued during follow-up, with GGT declining from the final value following treatment in the FIHS (43 and 77 IU/L, respectively), to within the normal range (24 and 24 IU/L, respectively) by the final follow-up visit. GGT levels also decreased in patient 107 but did not meet the criterion for a response based on GGT during the FIHS; however, levels continued to decrease during follow-up to within normal range (67 IU/L at end of FIHS to 18 IU/L at last follow-up). The same pattern was observed in patient 110, although normal range was not reached by the end of follow-up (88 IU/L at end of FIHS to 57 U/L at last follow-up visit). The GGT for patient 113 was in the normal range throughout the FIHS and the follow-up period.

#### Declining responders

The four declining responders with hepatic involvement had ApoA1 (patient 109) or AL (patients 111, 114, 115). GGT improved in patient 109 until 1-year post study, when it began to rise with a coincidental change in amyloid load on SAP scan from moderate to large. In patient 111 (who experienced clonal relapse) a large increase in GGT (from 643 U/L at  ~ 1-month post treatment to 1334 U/L 1 year later) was accompanied by a decrease in eGFR (56 mL/min/1.73 m^2^ ~ 1-month post treatment to 20 mL/min/1.73 m^2^ 1 year later) and a large increase in NT-proBNP levels (from 820 to 3214 ng/L); this patient died during follow-up. Patient 114 (who experienced clonal relapse) had improved GGT during the FIHS, which was maintained through the follow-up period; however, eGFR began to decline and NT-proBNP showed a slight rise to above the normal range from 2 years post study. In patient 115, GGT was in the normal range during the FIHS (21–22 U/L) and was stable during the follow-up period (16–22 IU/L), while eGFR and 6MWD declined and NT-proBNP increased.

### eGFR assessments in patients with renal involvement

**Fig. 5:**
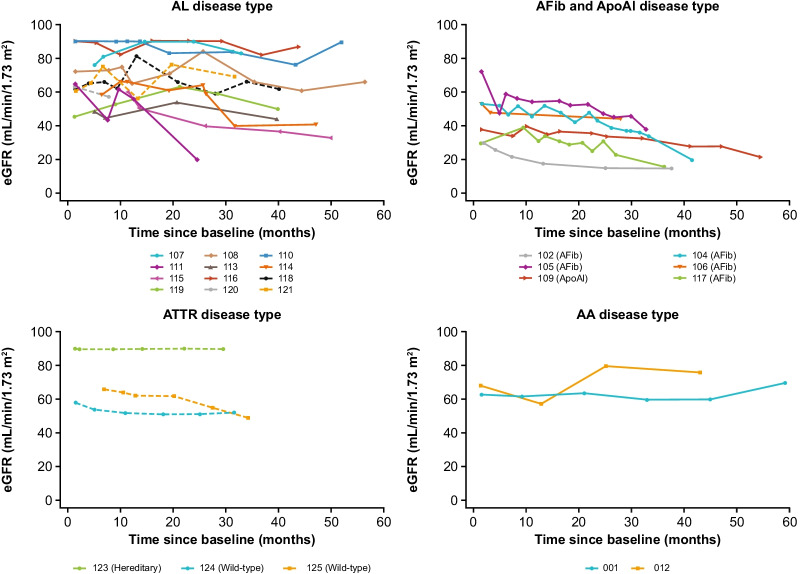
eGFR over time by patient. Dashed line, cardiac involvement; solid line, no cardiac involvement. Patients with values below the lower limit of quantification (< X) or above the upper limit of quantification (> X) have been plotted as values of X. Patient 102 reached end-stage renal disease and required double renal transplant after 3 years at which point their eGFR data were censored. AA, serum amyloid A; ApoAI, apolipoprotein A-I; AFib, fibrinogen-a alpha chain; AL, immunoglobulin light chain; ATTR, transthyretin; eGFR, estimated glomerular filtration rate

Patient-level renal function (eGFR) data are summarized by disease type in Fig. 5. Eleven patients with renal involvement were enrolled in the FIHS. Of these patients, four were defined as sustained responders, five as declining responders and one as a non-responder. The remaining patient received a non-therapeutic dose of miridesap/dezamizumab and was not included in the response classification.

#### Sustained responders

The four sustained responders with renal involvement had AA (patient 012) or AL (patients 108, 113, and 121). eGFR was stable over the follow-up period for patients 108 (61–84 mL/min/1.73 m^2^), 113 (44–54 mL/min/1.73 m^2^) and 012 (57–80 mL/min/1.73 m^2^). In patient 121 (with cardiac amyloid), eGFR fluctuated between 56 and 76 mL/min/1.73 m^2^ during follow-up.

#### Declining responders

The five declining responders with renal involvement all had AFib (patients 102, 104, 105, 106, 117). In patients 102, 105 and 117, eGFR declined during follow-up, with patient 102 reaching end-stage renal disease and requiring double renal transplant after 3 years; eGFR for patient 117 declined to 16 mL/min/1.73 m^2^ at the end of the observation period and dialysis was considered. In patient 104, eGFR was stable for 2 years post treatment, but then declined to < 30 mL/min/1.73 m^2^ by the end of follow-up. Patient 106 had stable eGFR during follow-up but decreasing 6MWD indicated a gradual decline in response.

### Cardiac assessments in patients with cardiac involvement

**Fig. 6:**
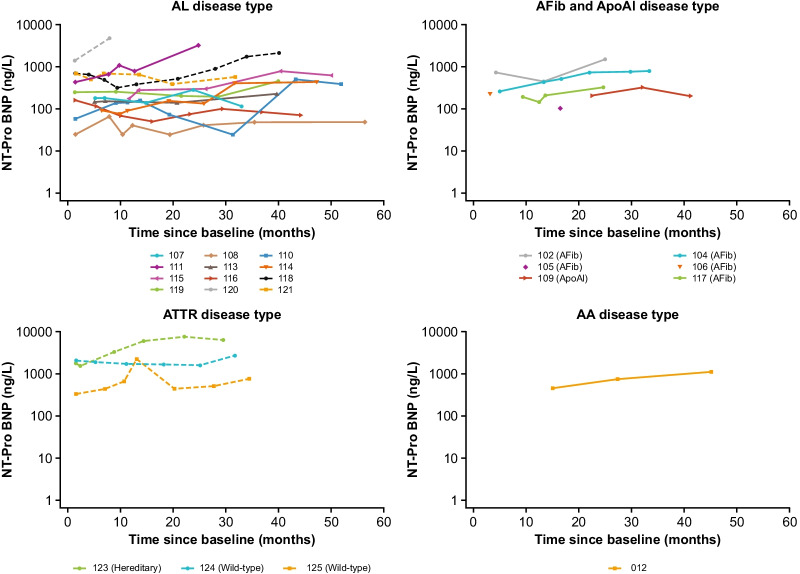
NT-proBNP over time by patient. Dashed line, cardiac involvement; solid line, no cardiac involvement. Patients with values below the lower limit of quantification (< X) or above the upper limit of quantification (> X) have been plotted as values of X. AA, serum amyloid A; ApoAI, apolipoprotein A-I; AFib, fibrinogen-a alpha chain; AL, immunoglobulin light chain; ATTR, transthyretin; NT-proBNP, N-terminal-pro B-type natriuretic peptide

Patient-level data showing NT-proBNP levels over time are presented by disease type in Fig. 6. Structural (LV septum and LV posterior wall thickness) and functional (E:E′ and LVEF) parameters are summarized for all patients in Additional file 6. Six patients (AL, n = 3; ATTR, n = 3 [1 hereditary, 2 wild-type]) with cardiac amyloid were enrolled in the FIHS. Of these patients, one was classified as a sustained responder and one as a declining responder. The remaining four patients were classified as non-responders (Additional file 2). All four of these patients showed increased or abnormal NT-proBNP levels during the follow-up period.

#### Sustained responders

The one sustained responder who had cardiac involvement (121) had a NT-proBNP level of 719 ng/L ~ 1 month after the last treatment session in the FIHS, and levels remained stable but above the normal range during follow-up (588 ng/L ~ 26 months after last treatment).

#### Declining responders

In the one declining responder who had cardiac involvement (118), NT-proBNP levels initially decreased after the FIHS from 499 ng/L ~ 1.5 months post treatment to 330 ng/L at ~ 4 months post treatment. Following this, NT-proBNP increased to abnormal levels ~ 27 months after the first dose (921 ng/L) and continued to increase up to the final follow-up visit (2170 ng/L).

### Cardiac assessments in patients without cardiac involvement

Of the patients without cardiac involvement, four declining responders had findings of note (patients 102 and 104 [renal involvement], 111 and 115 [hepatic involvement]). In these patients, NT-proBNP levels increased over the follow-up period. Further details on these patients are provided below.

### Imaging assessments of amyloid load by SAP scintigraphy

Changes in amyloid load for each patient (excluding those with ATTR) as determined by SAP scintigraphy scan are shown in Table 4.

**Table 4:** Imaging assessments (amyloid load based on SAP scan)

Subject number	Amyloid type	SAP scan output	1st value post diagnosis	Value at baseline*	Value at 1st follow-up	Value at 2nd follow-up	Value at 3rd follow-up	Value at 4th follow-up	Value at 5th follow-up	Value at 6th follow-up	Value at 7th follow-up
Sustained responders											
012	AA	Liver	Normal	Abnormal	–	–	–	–	–	–	–
		Spleen	Abnormal	Abnormal	Abnormal	–	–	–	–	–	–
		Kidney	Abnormal	Abnormal	Abnormal	–	–	–	–	–	–
		Adrenals	Abnormal	Abnormal	Abnormal	–	–	–	–	–	–
		Overall	Moderate	Moderate	Moderate	–	–	–	–	–	–
		Δ from prior visit	–	Stable	Stable	–	–	–	–	–	–
107	AL	Liver	Abnormal	Abnormal	Abnormal	Abnormal	–	–	–	–	–
		Spleen	Abnormal	Abnormal	Abnormal	Abnormal	–	–	–	–	–
		Kidney	Obscured	Obscured	–	–	–	–	–	–	–
		Adrenals	Obscured	Obscured	–	–	–	–	–	–	–
		Overall	Large	Moderate	Moderate	Moderate	–	–	–	–	–
		Δ from prior visit	–	–	Better	Stable	–	–	–	–	–
108	AL	Liver	Abnormal	Abnormal	Abnormal	–	Abnormal	–	Normal	Normal	–
		Spleen	Abnormal	Abnormal	Abnormal	Abnormal	Abnormal	–	Normal	Normal	–
		Kidney	Abnormal	–	Abnormal	–	Abnormal	Abnormal	Abnormal	Abnormal	Abnormal
		Adrenals	Obscured	–	–	–	Abnormal	–	Normal	Normal	–
		Overall	Large	Moderate	Large	Moderate	Small	Small	Small	Small	Small
		Δ from prior visit	–	Better	Better	Stable	Better	Stable	Stable	–	Stable
110	AL	Liver	Abnormal	Abnormal	Abnormal	Abnormal	Abnormal	Abnormal	–	–	–
		Spleen	Abnormal	Abnormal	Abnormal	Abnormal	Abnormal	Abnormal	–	–	–
		Kidney	Obscured	Abnormal	Obscured	Obscured	Normal	Normal	–	–	–
		Adrenals	Obscured	Obscured	–	–	Normal	Normal	–	–	–
		Overall	Large	Large	Large	Large	Large	Large	–	–	–
		Δ from prior visit	–	–	Stable	Stable	Stable	Stable	–	–	–
113	AL	Liver	Abnormal	–	Abnormal	Abnormal	–	–	–	–	–
		Spleen	Abnormal	–	–	Abnormal	–	–	–	–	–
		Kidney	Obscured	–	Equivocal	Equivocal	–	–	–	–	–
		Adrenals	Obscured	–	–	Normal	–	–	–	–	–
		Overall	Large	Large	Small	Small	–	–	–	–	–
		Δ from prior visit	–	Better	–	–	–	–	–	–	–
116	AL	Liver	Abnormal	Abnormal	Abnormal	Abnormal	Abnormal	Abnormal	Abnormal	–	–
		Spleen	Abnormal	Abnormal	Abnormal	Abnormal	Abnormal	Abnormal	Abnormal	–	–
		Kidney	Obscured	Abnormal	Abnormal	–	Abnormal	Normal	Obscured	–	–
		Adrenals	Obscured	Abnormal	–	–	Obscured	Normal	–	–	–
		Overall	Large	Large	Moderate	Large	Moderate	Large	Large	–	–
		Δ from prior visit	–	Stable	Better	Better	Stable	Stable	Stable	–	–
121	AL	Liver	–	–	–	Normal	–	–	–	–	–
		Spleen	Abnormal	Abnormal	Equivocal	Equivocal	–	–	–	–	–
		Kidney	–	–	–	Normal	–	–	–	–	–
		Adrenals	–	–	–	Normal	–	–	–	–	–
		Overall	Large	Moderate	–	–	–	–	–	–	–
		Δ from prior visit	–	Better	Better	–	–	–	–	–	–
Declining responders											
102	AFib	Liver	Normal	–	–	–	Normal	–	–	–	–
		Spleen	Abnormal	Abnormal	Abnormal	Abnormal	Abnormal	–	–	–	–
		Kidney	Abnormal	Abnormal	Abnormal	Abnormal	Abnormal	–	–	–	–
		Adrenals	Obscured	–	–	–	Obscured	–	–	–	–
		Overall	Moderate	Small	Small	Small	Small	–	–	–	–
		Δ from prior visit	–	Stable	–	Stable	Stable	–	–	–	–
104	AFib	Liver	Normal	Normal	Normal	Normal	–	–	–	–	–
		Spleen	Abnormal	Abnormal	Abnormal	Abnormal	–	–	–	–	–
		Kidney	Abnormal	Abnormal	Abnormal	Abnormal	–	–	–	–	–
		Adrenals	Obscured	Obscured	Normal	Obscured	–	–	–	–	–
		Overall	Small	Small	Small	Moderate	–	–	–	–	–
		Δ from prior visit	–	–	Stable	Stable	–	–	–	–	–
105	AFib	Liver	Normal	–	–	–	–	–	–	–	–
		Spleen	Normal	Abnormal	–	–	–	–	–	–	–
		Kidney	Normal	Abnormal	–	–	–	–	–	–	–
		Adrenals	Normal	–	–	–	–	–	–	–	–
		Overall	None	Small	–	–	–	–	–	–	–
		Δ from prior visit	–	Stable	–	–	–	–	–	–	–
106	AFib	Liver	Normal	–	Normal	–	–	–	–	–	–
		Spleen	Abnormal	–	Abnormal	–	–	–	–	–	–
		Kidney	Equivocal	Abnormal	Abnormal	–	–	–	–	–	–
		Adrenals	Normal	–	Normal	–	–	–	–	–	–
		Overall	Small	Moderate	Small	–	–	–	–	–	–
		Δ from prior visit	–	Stable	Stable	–	–	–	–	–	–
109	ApoA1	Liver	Abnormal	Abnormal	Abnormal	Abnormal	Abnormal	Abnormal	–	–	–
		Spleen	Abnormal	Abnormal	Abnormal	Abnormal	Abnormal	Abnormal	Abnormal	–	–
		Kidney	Obscured	Obscured	–	–	Obscured	Not functioning	Abnormal	–	–
		Adrenals	Obscured	–	–	–	Obscured	Normal	–	–	–
		Overall	Large	Large	Large	Large	Large	Large	Large	–	–
		Δ from prior visit	–	Worse	–	Stable	Worse	Stable	–	–	–
111	AL	Liver	Abnormal	Abnormal	–	Abnormal	–	–	–	–	–
		Spleen	Abnormal	Abnormal	–	Abnormal	–	–	–	–	–
		Kidney	Obscured	Obscured	–	–	–	–	–	–	–
		Adrenals	Obscured	–	–	–	–	–	–	–	–
		Overall	Large	Large	Large	–	–	–	–	–	–
		Δ from prior visit	–	Stable	–	–	–	–	–	–	–
114	AL	Liver	Abnormal	Abnormal	Abnormal	Abnormal	Abnormal	Abnormal	–	–	–
		Spleen	Abnormal	Abnormal	Abnormal	Abnormal	Abnormal	Abnormal	–	–	–
		Kidney	Abnormal	Abnormal	Abnormal	Abnormal	Abnormal	Abnormal	–	–	–
		Adrenals	Obscured	–	–	–	–	–	–	–	–
		Overall	Large	Moderate	–	Moderate	Moderate	Large	–	–	–
		Δ from prior visit	–	Better	Better	Worse	Stable	Better	–	–	–
115	AL	Liver	Normal	Abnormal	–	Abnormal	Abnormal	Abnormal	–	–	–
		Spleen	Abnormal	Abnormal	–	Abnormal	Abnormal	Abnormal	–	–	–
		Kidney	Obscured	–	–	–	Obscured	Obscured	–	–	–
		Adrenals	Obscured	–	–	–	Obscured	Obscured	–	–	–
		Overall	Large	–	–	Large	Large	Large	–	–	–
		Δ from prior visit	–	Stable	Stable	Stable	–	Better	–	–	–
117	AFib	Liver	–	–	–	–	–	–	–	–	–
		Spleen	Abnormal^†^	Abnormal^†^	Abnormal	Abnormal	–	–	–	–	–
		Kidney	Abnormal^†^	Abnormal^†^	Abnormal	Abnormal	–	–	–	–	–
		Adrenals	–	–	–	–	–	–	–	–	–
		Overall	Small^†^	Small^†^	Small	Small	–	–	–	–	–
		Δ from prior visit	–	–	Stable	–	–	–	–	–	–
118	AL	Liver	–	–	Normal	Normal	Normal	–	–	–	–
		Spleen	–	Abnormal	Abnormal	Abnormal	Abnormal	–	–	–	–
		Kidney	–	–	Normal	Normal	Normal	–	–	–	–
		Adrenals	–	–	Normal	Normal	Normal	–	–	–	–
		Overall	Moderate	Small	Small	Small	Small	–	–	–	–
		Δ from prior visit	–	Stable	Stable	Stable	Stable	–	–	–	–
Non-responder											
119	AL	Liver	Abnormal	Abnormal	Abnormal	Abnormal	Abnormal	–	–	–	–
		Spleen	Abnormal	Abnormal	Abnormal	Abnormal	Abnormal	–	–	–	–
		Kidney	Abnormal	Abnormal	Abnormal	–	Abnormal	–	–	–	–
		Adrenals	Obscured	–	–	–	–	–	–	–	–
		Overall	Large	Large	Large	Large	Large	–	–	–	–
		Δ from prior visit	–	Stable	Stable	Stable	–	–	–	–	–
120	AL	Liver	Normal	–	–	–	–	–	–	–	–
		Spleen	Abnormal	Abnormal	Abnormal	–	–	–	–	–	–
		Kidney	Abnormal	Abnormal	Abnormal	–	–	–	–	–	–
		Adrenals	Abnormal	Abnormal	Abnormal	–	–	–	–	–	–
		Overall	Moderate	Moderate	Moderate	–	–	–	–	–	–
		Δ from prior visit	–	–	Better	–	–	–	–	–	–
Non-therapeutic dose in FIHS											
001	AA	Liver	Normal	Normal	–	–	–	–	–	–	–
		Spleen	Abnormal	Abnormal	Abnormal	Abnormal	Abnormal	Abnormal	–	–	–
		Kidney	Abnormal	Abnormal	Abnormal	Abnormal	Abnormal	Abnormal	–	–	–
		Adrenals	Obscured	Obscured	–	–	–	–	–	–	–
		Overall	Moderate	Moderate	Small	–	–	Small	–	–	–
		Δ from prior visit	–	–	Better	–	Stable	Better	–	–	–

SAP scans not conducted in patients with ATTR (123, 124 and 125). Follow-up visits are presented in chronological order per parameter as given in the database. These visits will therefore occur at different times relative to baseline for each patient and parameter. Therefore, it is not possible to directly compare values at a particular visit between patients or between parameters within a patient

*The latest value in the database pre-baseline. The baseline date was defined as the date of first pharmacologically-active administration of dezamizumab (i.e. 200 mg in the session) in the FIHS. For subjects who only received a non-pharmacologically-active dose (i.e. < 200 mg in the session), their baseline date was defined as the date of first administration of a non-pharmacologically active dose of dezamizumab

^†^1st value post diagnosis and value at baseline were measured at the same study visit

AA, serum amyloid A; AFib, fibrinogen A alpha-chain; AL, immunoglobulin light chain; ApoA1, apolipoprotein A-I; ATTR, transthyretin; FIHS, first-in-human study; SAP, serum amyloid P

#### Sustained responders

In all sustained responders, overall amyloid load remained stable or decreased during follow-up. At the last follow-up visit, four patients had either small or moderate overall loads and two patients had large overall loads. Patient 121 did not have an SAP scan during follow-up.

#### Declining responders

In most declining responders, amyloid load remained stable (patients 102, 104, 106, 117 and 118) during follow-up. Although amyloid load was stable in these patients, they were classified as declining responders based on evidence for other response criteria (Table 3). In the remaining declining responders, amyloid load fluctuated between stable and worsening in patient 109 and between worsening and better in patient 114, with both changing from moderate load at end of treatment to large load at end of follow-up. In patient 115, fluctuations between stable and better amyloid load were seen, although load was still considered large at the final visit. Patient 105 did not have an SAP scan during follow-up, and data for patient 111 were limited, so changes in amyloid load during follow-up were not determined.

### FLC ratio in patients with AL experiencing clonal relapse

Kappa/lambda FLC ratio and levels are summarized in Additional file 7. Five patients with AL amyloidosis experienced clonal relapse; two were sustained responders (patients 110 [hepatic] and 113 [hepatic and renal]) and three were declining responders (patients 111, 114 [both hepatic] and 118 [cardiac]).

For the sustained responders, patient 110 experienced clonal relapse during the FIHS, ~ 2 months after first treatment session. During follow-up the kappa/lambda FLC ratio began to decline ~ 31 months after the last treatment session (1:0.04), indicating relapse; during the follow-up period treatment was administered. Patient 113 experienced a clonal relapse between treatment sessions during the FIHS, as evidenced by a high kappa/lambda FLC ratio (1:4.34) and underwent treatment, after which the ratio improved (1:1.14 by end of follow-up).

For the declining responders, narrative records indicated that patient 111 experienced clonal relapse in the FIHS after first treatment session and again near end of the observation period; ~ 1 year after the study, GGT and NT-proBNP significantly increased with a decline in eGFR, at which time the patient received treatment for clonal relapse. Narrative records indicated that patient 114 experienced clonal relapse during the FIHS and during the follow-up period; their amyloid load varied due to these relapses; treatment was administered. Patient 118 experienced clonal relapse during follow-up (evidenced by an increase in lambda FLC and fall in kappa/lambda FLC ratio to 1:0.14); treatment was administered.

### Serum amyloid A protein levels in patients with AA amyloidosis

In patient 001, who received a non-therapeutic dose of dezamizumab in the FIHS, serum amyloid A protein levels ranged from 4 to 18 mg/L. In patient 012, who was classified as a sustained responder (based on amyloid load), serum amyloid A proteins levels were 13 mg/L post diagnosis, decreased to 3 mg/L 1 month after baseline then increased to 12 mg/L at the last follow-up visit.

## Discussion

In systemic amyloidosis, the presence of amyloid is thought to be directly responsible for organ dysfunction. Effective treatments in AL amyloidosis targeting the underlying plasma cell dyscrasia have resulted in a steady increase in overall survival, with the median increasing from ~ 18 months in the 1980s to over 5 years in the 2010s [20, 21]. The patients with AL included in the follow-up study had demonstrated good responses to plasma cell therapies that limit the further production of amyloid prior to entering the FIHS. In the mid-2010s (the time of diagnosis of patients in this study) the median survival from diagnosis for ATTR-cardiac amyloidosis was between 25 and 41 months for hereditary and wild type disease, respectively [22]. There have been advances in the effective treatment of TTR amyloidosis with the regulatory agency approvals of the TTR stabilizer, tafamidis; approved in the early 2010s for TTR familial polyneuropathy and, the late 2010s for TTR cardiomyopathy [8]. No ATTR patients included in this study received tafamidis as this was approved for cardiomyopathy in the UK after the completion of this follow-up study. Alternative treatments for TTR, the TTR silencers patisiran [10] and inotersen [9] received regulatory approval for hereditary ATTR polyneuropathy in late 2010s and are undergoing clinical trials for ATTR cardiomyopathy. The development and approval of all these treatments has resulted in earlier and wider diagnosis, better clinical monitoring and improved standard of care in systemic amyloidosis, including supportive therapies for damaged organs and symptoms (e.g., kidney disease, hypertension) in all forms. However, currently, there are no approved treatments that actively remove existing amyloid deposits.

During the FIHS with miridesap/dezamizumab, evidence of amyloid removal in a substantial proportion of patients was observed, especially in the liver, spleen and kidney [16, 17]. Removal of amyloid may have the effect of ‘turning back time’ and resetting the patient’s clinical trajectory. The period of follow-up in the FIHS was limited; therefore, this long-term observational study provides extended follow-up to examine the outcomes for patients treated with miridesap/dezamizumab during the FIHS. It is acknowledged that this is a mixed cohort and the observations are considered preliminary.

In this observational, non-interventional follow-up study, we conducted a post hoc characterization of the natural history of patients according to their response to treatment with miridesap/dezamizumab. Of the 23 patients included in this analysis, seven and ten patients were considered sustained or declining responders, respectively (Table 5). Sustained responders mostly (n = 6) had AL amyloidosis (although two had clonal relapses), with the remaining patient having AA (n = 1) amyloidosis. Of the sustained responders with AL and an at diagnosis or post-diagnosis Mayo stage assessment, three were stage I, one was stage II.

**Table 5:** Summary of patient responses

Sustained responders n = 5	Sustained responders (with ongoing precursor protein deposition)* n = 2
Declining responders (with ongoing precursor protein deposition) n = 10	Non-responders n = 5

An additional patient not included in this table received a non-therapeutic dose in the FIHS so was not included in the classification of response

*Sustained responders with ongoing precursor protein deposition are defined as those with AFib (no patients), AApoAI (no patients) or AL and clonal relapse (patients 110 and 113)

AFib, fibrinogen A alpha-chain; AL, immunoglobulin light chain; ApoA1, apolipoprotein A-I; FIHS, first-in-human study

Three patients died during follow-up, two of whom were non-responders and had cardiac involvement (AL, n = 1; ATTR, n = 1), and the third who was a declining responder with hepatic involvement (AL). Time from diagnosis to death in the patients with cardiac involvement was 96.5 (AL) and 51.6 (ATTR) months, both of which are longer than predicted median survival times of 15.7 and 38.9 months, respectively [23]. Similarly, time from diagnosis to death in the patient with AL hepatic amyloidosis was longer than the median survival in a medical record review of 98 patients with this type of amyloidosis (121.0 vs 8.5 months) [24]. All patients classified as sustained responders (AL, n = 6; AA, n = 1) were alive at the end of the follow-up period/study termination. In comparison, a retrospective analysis of 230 patients with AL amyloidosis receiving cyclophosphamide/bortezomib/dexamethasone therapy, 55% of patients were predicted to survive at least 5 years [25]. In the sub-group of patients classified as cardiac stage II and IIIa, those with at least a very good partial response to treatment had an 84% survival probability at 36 months. Analysis of survival trends in patients with AL amyloidosis reveals that median overall survival has increased to more than 5 years in the past decade, as compared to a median of 18 months for patients diagnosed before 2005; primarily due to advances in therapies targeting plasma cells [20].

Amyloid load reduced or remained stable in most responders whether the response was sustained or declining, which suggests that amyloid load reduction or stabilization does not always correlate with functional improvement or preservation.

The most striking reductions in amyloid observed in the FIHS were in the liver. Nine of the ten patients with hepatic involvement had AL amyloidosis, and five showed a sustained response (two of whom achieved this response despite clonal relapse). In these sustained responders (patients 107, 108, 110, 113 and 116), who showed improved/stable liver function during follow-up based on GGT levels, the reduction or stabilization of amyloid load achieved in the FIHS was maintained during follow-up. This provides some encouragement that treatments able to remove amyloid can provide a lasting effect, but the clinical impact is hard to assess as hepatic involvement is not commonly a driver of clinical outcome.

In the FIHS, a reduction in total or renal amyloid was detected by SAP scintigraphy in 8/11 patients with renal involvement (five with AFib, two with AL and one with AA amyloidosis). No improvement in proteinuria was observed [16]. Long-term follow-up data from this study show that the reduction in amyloid load seen in the FIHS was mostly maintained; however, all but one of the patients with AFib experienced a gradual decline in eGFR typical of this form of amyloidosis, and it is not apparent that the treatment had any impact on the rate of eGFR decline. The patient with AA amyloidosis had stable renal function over the period of follow-up.

Patients with hereditary forms of amyloidosis, for example, ApoAI and AFib [1], experience ongoing deposition of amyloid protein. As such, achieving sustained suppression of amyloid load in these patients is challenging. In this study, the patient with ApoAI experienced improved amyloid load during the FIHS, which fluctuated between stable and worse over follow-up. All five patients with AFib had hepatic involvement, and reductions in amyloid load were seen during the FIHS. However, during follow-up four of these patients experienced declining renal function that resulted in end-stage renal disease in one patient and consideration of dialysis in another.

The findings of this study should be considered in light of the study limitations. This was a descriptive study in a small (n = 23) population of patients with amyloidosis of varying aetiologies. Since patients with cardiac involvement were excluded from the initial phase of the FIHS for safety reasons, the study population included in this analysis is not necessarily representative of the general population of patients with amyloidosis. A phase 2 study of miridesap/dezamizumab in patients with cardiac involvement has been completed to address this patient population [26]. In each patient, assessments were conducted at different times relative to the baseline date, precluding comparisons between patients at a specific visit; however, this was not the intention of the study. In addition, patients may have more post-baseline values for some parameters than others; therefore, it is not possible to compare parameters at a specific visit number or time point within a patient. Another limitation relates to the subjective and post hoc nature in which the response classification was performed. However, given the heterogeneity of patients and lack of consistency in data among and within patients, we believe that this approach was appropriate and ensured that the overall patient profile was considered in determining the status of response. Further, since these were not included in the National Amyloidosis Centre database accessed, this study is limited by a lack of patient-reported outcome measures, these should be included in any future planned studies.

In conclusion, the long-term characterization of patients with amyloidosis who received miridesap/dezamizumab presented here may be useful in informing future investigations, particularly regarding insight into whether active removal of amyloid deposits affects disease progression. Patients with AL showing a clear response (as evidenced by reduction in organ amyloid on SAP scans in the FIHS) generally demonstrated a sustained or continued improvement in response through the follow-up period, unless they entered clonal relapse. In patients with hereditary-type amyloidosis, in whom the precursor protein is produced continually, reductions in amyloid deposits in kidney/spleen were seen in the FIHS but renal function continued to decline in most patients. Although no further development of the anti-SAP treatment in systemic amyloidosis is planned, we could speculate that longer-term therapeutic interventions driving active removal of amyloid deposits, particularly in patients with AL amyloidosis, may be beneficial.

## Supplementary Information

**Additional file 1:** Full study protocol.

**Additional file 2:** Supplementary results.

**Additional file 3: Table S1**. ECOG performance status.

**Additional file 4: Table S2**. NYHA Class.

**Additional file 5: Table S3**. 6MWD (m).

**Additional file 6: Table S4**. Cardiac assessments (grey shading denotes subjects with cardiac involvement).

**Additional file 7: Table S5**. Involved free light chains (iFLC) in mg/L at follow-up visits in patients with AL amyloid.

## Data Availability

The datasets used during the current study are available from www.clinicalstudydatarequest.com on reasonable request.
