# Supplementary material for: An observational, non-interventional study for the follow-up of patients with amyloidosis who received miridesap followed by dezamizumab in a phase 1 study
Source: Orphanet J Rare Dis. 2022 Jul 9;17:259. doi: 10.1186/s13023-022-02405-7 (PMC9271258; doi:10.1186/s13023-022-02405-7)
Supplement: Supplementary file 1 — Additional file 1: Full study protocol. [file 13023_2022_2405_MOESM1_ESM.pdf]

Division: Worldwide Development
Information Type: Worldwide Epidemiology Study Protocol

**Title:**	An observational, non-interventional Study for the follow-up of subjects receiving GSK2398852

**Compound Number:**	GSK2398852 + GSK2315698
**Development Phase**	I
**Effective Date:**	21-MAR-2014

Subject: GSK2398852, GSK2315698, systemic amyloidosis, patient, clinical, SAP, observational.

Author(s): PPD (Clinical Pharmacology Sciences and Study Operations); PPD (Academic DPU); PPD (Clinical Pharmacology Sciences and Study Operations); PPD (QSci); PPD (National Amyloidosis Centre, London); PPD (National Amyloidosis Centre, London)

Copyright 2014 the GlaxoSmithKline group of companies. All rights reserved. Unauthorised copying or use of this information is prohibited

TABLE OF CONTENTS

PAGE

1. LIST OF ABBREVIATIONS 3

2. ABSTRACT 7

3. AMENDMENTS AND UPDATES 7

4. RATIONALE AND BACKGROUND 8
   4.1. Background 8
      4.1.1. Amyloid and Amyloidosis 8
      4.1.2. Serum amyloid P component (SAP) 8
   4.2. Rationale 9

5. RESEARCH QUESTION AND OBJECTIVE(S) 10

6. RESEARCH METHODS 10
   6.1. Study Design 10
   6.2. Setting 10
   6.3. Variables 10
      6.3.1. Confounders and effect modifiers 11
   6.4. Data sources 12
   6.5. Study size 12
   6.6. Data management 12
   6.7. Data analysis 12
      6.7.1. Renal and liver function laboratory parameters, haemoglobin, cardiac biomarkers, functional status measures, Mayo disease stage, echocardiogram parameters and FLC measures 12
      6.7.2. Amyloid load (SAP scan) 13
      6.7.3. Survival data 13
   6.8. Quality control 13
   6.9. Limitations of the research methods 14
   6.10. Study closure 14
      6.10.1. Retention of Study Data 14

7. PROTECTION OF HUMAN SUBJECTS 14
   7.1. Ethical approval and subject consent 14
      7.1.1. Informed Consent 15
   7.2. Ethical Study Conduct 15
      7.2.1. Protocol Amendments 15
      7.2.2. Monitoring 15
   7.3. Subject confidentiality 16

8. PLANS FOR DISSEMINATING AND COMMUNICATING STUDY RESULTS 16
   8.1. Study reporting and publications 16

9. REFERENCES 17

1. LIST OF ABBREVIATIONS

CRP	C-reactive protein
ECOG	Eastern Cooperative Oncology Group
FLC	Free Light Chain
GCP	Good Clinical Practice
GSK	GlaxoSmithKline
ICH	International Conference on Harmonisation
IEC	Independent Ethics Committee
mAb	Monoclonal Antibody
NYHA	New York Heart Association
SAP	Serum Amyloid P Component
UK	United Kingdom

Trademark Information

Trademarks of the GlaxoSmithKline group of companies
NONE

Trademarks not owned by the GlaxoSmithKline group of companies
None

SPONSOR SIGNATORY:

PPD

PPD

Dr Duncan Richards
VP Clinical Head, Ac DPU

Da[illegible]

SPONSOR INFORMATION PAGE

Project Identifier: SAP115970

Sponsor Legal Registered Address:

GlaxoSmithKline Research & Development Limited
980 Great West Road
Brentford
Middlesex, TW8 9GS
UK

Sponsor Contact Address

GlaxoSmithKline Research & Development Limited
Iron Bridge Road
Stockley Park West, Uxbridge, Middlesex, UB11 1BU, UK
Telephone: PPD [redacted]

In some countries, the clinical trial sponsor may be the local GlaxoSmithKline affiliate company (or designee). Where applicable, the details of the Sponsor and contact person will be provided to the relevant regulatory authority as part of the clinical trial submission.

Sponsor Medical Monitor Contact Information:

Role	Name	Day Time Phone Number	After-hours Phone Number	Fax Number	GSK Address
Primary Medical Monitor	Dr. PPD [redacted]	PPD [redacted]	[redacted]	[redacted]	GlaxoSmithKline, Clinical Unit Cambridge Addenbrooke's Hospital, Box 128, Hills Rd, Cambridge CB2 2GG, UK
Secondary Medical Monitor	Dr. PPD [redacted]	[redacted]	[redacted]	[redacted]	5 Moore Drive N2.3209 Research Triangle Park NC.27709

Regulatory Agency Identifying Number(s): Not Applicable

INVESTIGATOR PROTOCOL AGREEMENT PAGE

- I confirm agreement to conduct the study in compliance with the protocol.
- I acknowledge that I am responsible for overall study conduct. I agree to personally conduct or supervise the described clinical study.
- I agree to ensure that all associates, colleagues and employees assisting in the conduct of the study are informed about their obligations. Mechanisms are in place to ensure that site staff receives the appropriate information throughout the study.

Investigator Name: ______________________________

____ ____________________

Investigator Signature Date

2. ABSTRACT

Rationale

The action of the anti-SAP monoclonal Antibody [mAb] (GSK2398852) is expected to lead to clearance of amyloid deposits. This has the potential to impact the long term prognosis of patients with systemic amyloidosis. Systemic amyloidosis is a rare disease and the number of subjects administered with GSK2398852 will be small, it is therefore especially important to collect comprehensive information on all subjects exposed. The purpose of this study is to collect natural history data from patients with systemic amyloidosis who have been administered with the anti-SAP antibody (GSK2398852 given with the SAP depleter GSK2315698) in GSK-sponsored clinical trial SAP115570, in order to inform the design of future clinical studies.

Objective(s)

The objective of the study is to collect comprehensive natural history information on subjects who have received GSK2398852 and GSK2315698, in order to inform the design of future clinical studies. This study will track overall clinical status and key organ function.

Study Design

The study will be an observational natural history study. Subjects who have received GSK2398852 and GSK2315698 in GSK-sponsored clinical trial SAP115570 will be invited to participate. Subjects will have completed follow up assessments as part the interventional study. If they agree to participate, subjects will undergo their usual clinical visits, and relevant data related to overall clinical status and key organ function will be collated approximately every 6 months for up to 5 years post last dose. In order to provide context for the data, the same information, where available, will also be collated from diagnosis until entry in to this study. Subjects will not be asked to undertake any additional visits or investigations as a result of participation in this observational study.

3. AMENDMENTS AND UPDATES

Version No	Date	Section of study protocol	Amendment or update	Reason
00 - Original	N/A	N/A	N/A	N/A

4. RATIONALE AND BACKGROUND

4.1. Background

4.1.1. Amyloid and Amyloidosis

Amyloidosis is a clinical disorder caused by extracellular deposition of amyloid in visceral parenchyma, connective tissues and blood vessel walls damaging their structure and function [Pepys, 2006]. In systemic amyloidosis any tissue or organ may be affected except the cerebral parenchyma. In local amyloidosis the deposits are confined to a single organ or tissue type.

Amyloid deposits are largely composed of amyloid fibrils, which are abnormal, insoluble fibrous structures, composed of normally soluble autologous proteins which have undergone misfolding, losing their stable native structure and/or assembly and then aggregating as stable fibres with a characteristic cross-β core [Sunde, 1997].

The amyloid fibril precursor proteins in systemic amyloidosis are derived from the circulation while in local amyloidosis the fibril protein may be produced locally. Although more than 25 different proteins have been identified as forming amyloid fibrils in different types of amyloidosis and amyloid deposits, the fibrils always share the common cross-β core structure and similar ultrastructural fibrillar morphology.

All amyloid deposits are rich in heparin and dermatan sulphate proteoglycans and glycosaminoglycan chains, tightly associated with the fibrils [Nelson, 1991]. Also all amyloid deposits always contain the normal plasma glycoprotein, serum amyloid P component (SAP), as a result of its specific calcium dependent binding to all types of amyloid fibrils [Pepys, 1979] including synthetic fibrils created in vitro from isolated pure fibril precursor proteins [Pepys, 1997].

In systemic amyloidosis, progressive amyloid deposition leads inexorably to organ dysfunction and failure, and is almost always fatal. In the most common type of systemic amyloidosis, monoclonal immunoglobulin light chain (AL) disease (sometimes previously referred to as primary amyloidosis), median survival is about 12-15 months without treatment. In reactive systemic (AA) amyloidosis (sometimes previously referred to as secondary amyloidosis), a complication of chronic inflammatory diseases, and in most forms of hereditary amyloidosis, patients may survive for 5-10 years. Treatments to reduce the abundance of the respective fibril precursor proteins can prolong survival for several years but these may not be available in all types of amyloidosis, are often toxic, and rarely cure the primary disease. Although these interventions may arrest amyloid deposition and in some patients regression of the deposits then ensues, no treatments currently exist which specifically target amyloid for elimination [Pepys, 2006].

4.1.2. Serum amyloid P component (SAP)

Human SAP, is a normal, non-fibrillar, constitutive plasma glycoprotein circulating at ~20-40 mg/L which, together with C-reactive protein (CRP) forms the pentraxin family of proteins [Pepys, 1997]. Although SAP is an acute phase protein in mice [Pepys, 1979]

and some other rodents [Pepys, 1997], its circulating concentration is stable in man in health and disease, including amyloidosis, except in individuals with liver failure since SAP is uniquely synthesised by hepatocytes [Pepys, 1978; Nelson, 1991]. A total of about 50-100 mg of SAP is present in the combined plasma and extravascular compartments both of normal individuals and patients with diseases other than amyloidosis [Hawkins, 1990]. SAP is also a normal constituent of the extracellular matrix located on the microfibrillar mantle of elastic fibres throughout the body [Breathnach, 1981] and in the *lamina rara interna* of the glomerular basement membrane [Dyck, 1980]. However the amounts of SAP in these normal tissue sites are extremely small compared to the quantity of fluid phase SAP. In patients with amyloidosis, SAP is also specifically concentrated in the amyloid deposits, and in an individual with extensive systemic amyloidosis there may be as much as 20,000 mg of SAP in the amyloid deposits, reversibly bound to the fibrils and in equilibrium with the fluid phase SAP pool.

The pentraxins are phylogenetically ancient proteins and their sequences, native fold, molecular assembly and capacity for calcium dependent ligand binding are highly conserved across species. However, their circulating concentration, behaviour as acute phase proteins, glycosylation, fine ligand specificity and capacity for secondary effects after ligand binding, including precipitation, aggregation and complement activation, vary widely, even between closely related species [Baltz, 1982; Pepys, 1983]. Their normal physiological roles are therefore not clearly defined and their biological properties are controversial.

The most robust observations on the function of SAP are that its binding to various ligands, including amyloid fibrils, stabilises them substantially and protects them from proteolytic cleavage [Tennent, 1995]. SAP binding also promotes amyloid fibrillogenesis *in vitro* [Hamazaki, 1995; Myers, 2006]. Although mouse SAP binds much more weakly to amyloid fibrils than does human SAP [Baltz, 1986; Hawkins, 1988; Herbert, 2002], SAP knockout mice show delayed and reduced formation of amyloid deposits [Botto, 1997] and SAP thus seems to contribute to the pathogenesis and/persistence of amyloid in vivo.

4.2. Rationale

The action of the anti-SAP mAb (GSK2398852) is expected to lead to clearance of amyloid deposits. This has the potential to impact the long term prognosis of patients with systemic amyloidosis. Systemic amyloidosis is a rare disease and the number of subjects administered GSK2398852 will be small, it is therefore especially important to collect comprehensive information on all subjects exposed. The purpose of this study is to collect natural history data from patients with systemic amyloidosis who have been administered with the anti-SAP antibody (GSK2398852 given with the SAP depleter GSK2315698) in GSK-sponsored clinical trial SAP115570, in order to inform the design of future clinical studies. Subjects in this study will have completed follow up assessments as part of the interventional study.

5. RESEARCH QUESTION AND OBJECTIVE(S)

The objective of the study is to collect comprehensive natural history information on subjects who have received GSK2398852 and GSK2315698, in order to inform the design of future clinical studies. This study will track overall clinical status and key organ function.

6. RESEARCH METHODS

6.1. Study Design

The study will be an observational natural history study. Subjects who have received GSK2398852 and GSK2315698 and completed follow up in GSK-sponsored clinical trial SAP115570 will be invited to participate. If they agree to participate, subjects will undergo their usual clinical visits, and relevant data related to overall clinical status and key organ function will be collated approximately every 6 months for up to 5 years post last dose. In order to provide context, the same information, where available, will be collated from diagnosis until entry in to this study. Subjects will not be asked to undertake any additional visits or investigations as a result of participation in this observational study.

6.2. Setting

Patients who have received GSK2398852 and GSK2315698 and completed follow up as part of the GSK-sponsored first time in human study SAP115570, conducted in the UK, will be invited to participate.

Patients from other centres and other studies as they are conducted may be recruited into the study without need for an amendment being made to this protocol.

The population recruited to this study will reflect the inclusion/exclusion criteria for the interventional study.

This is a non-interventional study and no study procedures will be performed. Information relating to the clinical course of patients is already being collected and databased as part of the routine standard of care for these patients. Data for this study will be obtained by extracting anonymised information from this database.

6.3. Variables

Data will be collated approximately every 6 months. These data points include parameters collected as part of routine standard of care for amyloidosis patients, and may include (but not limited to):

- disease status
    - SAP scan result
    - Mayo disease stage
    - free light chain (FLC) data (AL patients only)
    - serum immunoglobulin (AL patients only)

  - protein (serum and urine; AL patients only)
  - serum amyloid A (AA patients only)
  - cardiac biomarkers
  - renal function laboratory parameters
  - liver function laboratory parameters
  - liver elastography
  - echocardiogram parameters
  - haemoglobin
- disease type (e.g. AL, AA)
- survival outcome
- functional status
  - Eastern Cooperative Oncology Group (ECOG)
  - New York Heart Association (NYHA)
  - 6 minute walk test (6MWT)
- vital signs.

Note, the following data are not collected in GSK-sponsored clinical trial SAP115570: Mayo disease stage, FLC, echocardiogram, ECOG, NYHA and 6MWT.

Summary information on amyloid-specific therapies received during the follow-up period will also be collated where available, including:

- chemotherapeutic agents (AL patients) - number of cycles, treatment course stop/start date
- anti-inflammatory therapies (AA patients) - treatment stop/start date
- other amyloid specific therapies (including investigational therapies) - treatment stop/start date
- organ transplantion – organ, transplant date.

Dose information for these amyloid-specific therapies will not be collated.

GSK2398852 dosing information from previous studies in which patients have participated will be included in the assessment of the follow-up data from this study. GSK2315698 dosing information will not be included.

No investigational drug will be administered under this clinical protocol.

6.3.1. Confounders and effect modifiers

The population in this study will reflect that recruited to the interventional studies and cannot be assumed to represent the systemic amyloid population as a whole. Data will generally be presented as subject narratives.

Subjects involved in the study may be exposed to additional medications for the treatment of amyloidosis, or may receive organ transplantation. Therefore, any change in clinical course may be related either to administration of GSK2398852 or GSK2315698 or another treatment regimen.

6.4. Data sources

Informed consent forms, subject files, information contained within the database and the summary records of amyloid-specific therapies are considered to be source data for this study.

6.5. Study size

This study is descriptive in nature, and no formal hypotheses will be tested. The study will enrol patients from clinical study SAP115770 who were administered with GSK2398852 and GSK2315698. Patients from other centres and other studies as they are conducted may be recruited into the study without need for an amendment being made to this protocol.

6.6. Data management

Data will be obtained from existing databases at the clinical assessment site that the patient visits as part of their normal clinical care and summary records of amyloid-specific therapies received.

An excel extraction of the data will be performed by the clinical assessment site, personally identifiable data will be removed and replaced with the subject identifiers from the GSK interventional studies. A summary record of amyloid-specific therapies received will also be provided.

An extract of the database will occur at approximately 6-monthly intervals for up to 5 years after the last subject is recruited into the study and dosed.

6.7. Data analysis

Data will be reviewed on an ongoing basis approximately every six months as data is extracted from the clinical site database. A detailed description of all planned analyses will be provided in the reporting and analysis plan.

6.7.1. Renal and liver function laboratory parameters, haemoglobin, cardiac biomarkers, functional status measures, Mayo disease stage, echocardiogram parameters and FLC measures

Individual plots over time will be presented for available data from diagnosis to up to 5 years post last administration of GSK2398852.

Individual plots of changes over time will be presented for available data from diagnosis (or earliest available data) to up to 5 years post last administration of GSK2398852.

Individual plots of changes over time will also be presented for available data from day of first administration of GSK2398852 to up to 5 years post last administration of GSK2398852.

6.7.2. Amyloid load (SAP scan)

Individual plots over time of whole body amyloid load will be presented for available data from diagnosis (or earliest available data) to up to 5 years post last administration of GSK2398852. Individual plots over time of whole body amyloid load will also be presented for available data from day of first administration of GSK2398852 to up to 5 years post last administration of GSK2398852.

6.7.3. Survival data

Kaplan-Meier survival plots relative to day of diagnosis may be presented if data permit over all patients and also by disease type, by Mayo stage at diagnosis, by overall amyloid load (SAP scan score) at diagnosis and by highest received GSK2398852 dose (with lower dose groups combined). Further subgroups may also be explored.

Kaplan-Meier survival plots relative to day of first administration of GSK2398852 may also be presented if data permit over all patients and also by disease type, by Mayo stage at pre-dose, by overall amyloid load (SAP scan score) at pre-dose and by highest received GSK2398852 dose (with lower dose groups combined). Further subgroups may also be explored.

Median and 25th and 75th percentile survival times relative to diagnosis and relative to first administration of GSK2398852 may also be estimated over all patients and by subgroups if data permit together with corresponding 95% CIs.

Further exploratory analyses using a Cox regression model may also be performed, if data permit, to further explore the impact of selected baseline and/or time-dependent variables on survival time from diagnosis. Baseline and/or time-dependent variables for exploration may include age, Mayo stage, amyloid load (overall and/or organ-specific), cardiac biomarkers, renal function, liver function, functional status and echocardiogram parameters. Further baseline-only variables for exploration may include disease type. Further time-dependent only variables for exploration may include GSK2398852 administration (and/or dose level) and other amyloid-specific therapies such as chemotherapy, anti-inflammatory therapies, organ transplantation.

A detailed description of all planned analyses of survival data will be provided in the reporting and analysis plan.

6.8. Quality control

To ensure compliance with GCP and all applicable regulatory requirements, the investigators and associated institutions will permit study-related audits, IEC review, and regulatory inspections, providing direct access to source data and documents.

GSK, or external regulatory agencies may at any time during or after completion of the study conduct a GCP audit. Prior notice will be given to each site selected in advance of a planned GCP audit.

The investigator should promptly notify GSK of any audits scheduled by any regulatory authorities and promptly forward copies of any audit reports received to GSK.

6.9. Limitations of the research methods

It is anticipated that administration of anti-SAP mAb could alter the prognosis for patients with systemic amyloid. This study will provide natural history data for subjects who have received GSK2315698 and GSK2398852 in an early clinical development study. The population and doses administered will be diverse reflecting the early development stage. The number of subjects will also be small. Within the period during which data are collected it is likely that subjects will receive other therapies for systemic amyloid which will limit the interpretation of impact on clinical course.

6.10. Study closure

Upon completion or termination of the study, the monitor will conduct site closure activities with the investigator or site staff (as appropriate), in accordance with applicable regulations, ICH GCP, and GSK standard operating procedures.

GSK reserves the right to temporarily suspend or terminate the study at any time for reasons including (but not limited to) safety or ethical issues. If GSK determines that such action is required, GSK will discuss the reasons for taking such action with the investigator or head of the medical institution (where applicable). When feasible, GSK will provide advance notice to the investigator or head of the medical institution of the impending action.

6.10.1. Retention of Study Data

The investigator is required to maintain all study documentation, including regulatory documents and signed informed consent forms, for a period of at least two years following approval date of a New Drug Application for the drug or until 15 years after completion of the study, whichever is later.

During the study, the investigator must make study data accessible to GSK, the Independent Ethics Committee (IEC), and the FDA and other regulatory agencies. A file for each subject must be maintained that includes the signed informed consent form and copies of all source documentation related to that subject. The investigator must ensure the availability of source documents from which the data provided to GSK were derived.

7. PROTECTION OF HUMAN SUBJECTS

7.1. Ethical approval and subject consent

The study will be conducted in accordance with Good Clinical Practice (GCP), all applicable subject privacy requirements, and the ethical principles that are outlined in the Declaration of Helsinki 2008, including, but not limited to:

- Independent Ethics Committee (IEC) review and approval of study protocol and any subsequent amendments

- Subject informed consent
- Investigator reporting requirements

GSK will provide full details of the above procedures, either verbally, in writing, or both. Written informed consent must be obtained from each subject prior to participation in the study.

7.1.1. Informed Consent

The International Conference on Harmonisation (ICH) principles of informed consent in the Declaration of Helsinki, in ICH E6(R1), will be implemented before any data are collected.

A signed informed consent form shall be obtained from each subject prior to entry into the study. The investigator is responsible for obtaining written informed consent from the subject after adequate explanation of the aims, methods of the study and before any data is extracted. Information should be given in both oral and written form whenever possible and deemed appropriate by the IEC. Subjects will also be asked to consent to allow GSK, GSK representatives, and any external regulatory auditor to review their medical records to confirm compliance with GCP.

The acquisition of informed consent should be documented in the subject's medical record, and the informed consent form should be signed and personally dated by the subject and by the person who conducted the informed consent discussion (not necessarily by the investigator). The original signed informed consent form should be retained in the investigator site file, and a copy of the signed consent should be provided to the subject prior to participation in the trial.

The subjects will be informed that they may withdraw from the study at any time without prejudice to further treatment. They will receive all information that is required by local regulations and ICH guidelines.

7.2. Ethical Study Conduct

7.2.1. Protocol Amendments

No amendments to the protocol will be implemented prior to agreement from GSK and prior to approval from the appropriate authorities where required.

7.2.2. Monitoring

Remote and on-site monitoring will be conducted to ensure consistent and meaningful communication with site personnel. The study monitor assigned to the site will be the first line of communication and will have great influence over the site's subject follow-up.

Approximately every six months, follow-up data collected as part of patients' routine care will be sent to GSK. Once the data have been received in-house, they will be

checked for obvious errors and omissions, and any queries will be communicated to the site until resolved.

The study monitor will review the progress of the study on a regular basis to ensure adequate and accurate data collections. At each study monitoring visit to site, the investigator will make available all records pertaining to the study including completed consent forms. Significant or relevant communications with GSK should be documented by the site and retained for the study file.

7.3. Subject confidentiality

The investigator must ensure that the subject's anonymity is maintained. On all study documentation, with the exception of the consent form and subject identification logs, each subject will be identified only by his or her unique identification code(s) from the previous GSK-sponsored clinical study and will not be referred to by name.

All records will be kept in a secure storage area with limited access. Clinical information will not be released without the written permission of the subject (or the subject's legal guardian), except as necessary for monitoring and auditing by GSK, its designee, the United States Food and Drug Administration (FDA) or other regulatory agencies, or the IEC.

The investigator and all employees and co-workers involved with this study may not disclose or use for any purpose other than performance of the study any data, record, or other unpublished, confidential information disclosed to those individuals for the purpose of the study. Prior written agreement from GSK or its designee must be obtained for the disclosure of any said confidential information to other parties.

Subject identifiers will not be provided to GSK, and GSK will have no ability to link the unique study number to the subject name or medical record number. All data analysis and summaries will be based on the subject's unique subject number from the previous GSK-sponsored clinical study, which is not linked to identifiable information.

8. PLANS FOR DISSEMINATING AND COMMUNICATING STUDY RESULTS

8.1. Study reporting and publications

A detailed narrative for each subject will be developed related to their exposure to GSK2398852.

Upon completion of the study, a final study report will be developed. The report will include narratives for each subject. In addition, GSK may develop and submit a manuscript based on the study results to a peer-reviewed journal for publication purposes.

9. REFERENCES

Baltz ML, Caspi D, Evans DJ, Rowe IF, Hind CRK, Pepys MB. Circulating serum amyloid P component is the precursor of amyloid P component in tissue amyloid deposits. *Clin. Exp. Immunol.* 1986;66:691-700

Baltz ML, de Beer FC, Feinstein A, Munn EA, Milstein CP, Fletcher TC, March JF, Taylor J, Bruton C, Clamp JR, Davies AJS and Pepys MB. Phylogenetic aspects of C-reactive protein and related proteins. *Ann. N.Y. Acad. Sci*., 1982;389:49-75

Botto M, Hawkins PN, Bickerstaff MC, Herbert J, Bygrave AE, McBride A, Hutchinson WL, Tennent GA, Walport MJ, Pepys MB. Amyloid deposition is delayed in mice with targeted deletion of the serum amyloid P component gene. *Nat Med*. 1997;3(8):855-9.

Breathnach SM, Melrose SM, Bhogal B, de Beer FC, Dyck RF, Tennent G, Black MM, Pepys MB. Amyloid P component is located on elastic fibre microfibrils in normal human tissue. *Nature*. 1981;293(5834):652-4

Dyck RF, Evans DJ, Lockwood CM, Rees AJ, Turner D, Pepys MB. Amyloid P-component in human glomerular basement membrane. Abnormal patterns of immunofluorescent staining in glomerular disease. Lancet. 1980;2(8195 pt 1):606-9

Hamazaki H. Amyloid P component promotes aggregation of Alzheimer's β-amyloid peptide. *Biochem. Biophys. Res. Commun.*, 1995;211:349-353

Hawkins PN, Myers MJ, Epenetos AA, Caspi D, Pepys MB. Specific localization and imaging of amyloid deposits *in vivo* using ^{123}I-labeled serum amyloid P component. *J. Exp. Med.* 1988;167:903-913

Hawkins PN, Wootton R, Pepys MB. Metabolic studies of radioiodinated serum amyloid P component in normal subjects and patients with systemic amyloidosis. *J Clin Invest.* 1990;86(6):1862-9.

Herbert J, Hutchinson WL, Carr J, et al. Influenza virus infection is not affected by serum amyloid P component. *Mol. Med.* 2002;8:9-15

Myers SL, Jones S, Jahn TR, Morten IJ, Tennent GA, Hewitt EW and Radford SE. A systematic study of the effect of physiological factors on β_2-microglobulin amyloid formation at neutral pH. *Biochemistry*. 2006;45:2311-2321

Nelson SR, Lyon M, Gallagher JT, Johnson EA and Pepys MB. Isolation and characterization of the integral glycosaminoglycan constituents of human amyloid A and monoclonal light-chain amyloid fibrils. *Biochem. J.*,1991;275:67-73

Pepys MB and Baltz ML. Acute phase proteins with special reference to C-reactive protein and related proteins (pentaxins) and serum amyloid A protein. Adv. Immunol. 1983;34:141-212.

Pepys MB, Booth DR, Hutchinson WL, Gallimore JR, Collins PM and Hohenester E. Amyloid P component. A critical review. Amyloid: *Int. J. Exp. Clin. Invest*. 1997;4:274-295.

Pepys MB, Dash AC, Markham RE, Thomas HC, Williams BD, Petrie A. Comparative clinical study of protein SAP (amyloid P component) and C-reactive protein in serum. *Clin Exp Immunol* 1978;32:119-124

Pepys MB, Dyck RF, de Beer FC, Skinner M and Cohen AS. Binding of serum amyloid P component (SAP) by amyloid fibrils. *Clin. Exp. Immunol*. 1979;38: 284-293

Pepys MB. Amyloidosis. *Annu. Rev. Med*. 2006;57:223-241.

Sunde M, Serpell LC, Bartlam M, Fraser PE, Pepys MB and Blake CCF. Common core structure of amyloid fibrils by synchrotron X-ray diffraction. *J. Mol. Biol*. 1997; 273:729-739.

Tennent GA, Lovat LB and Pepys MB. Serum amyloid P component prevents proteolysis of the amyloid fibrils of Alzheimer's disease and systemic amyloidosis. *Proc. Natl. Acad. Sci. USA*. 1995;92:4299-4303
