# Supplementary material for: An observational, non-interventional study for the follow-up of patients with amyloidosis who received miridesap followed by dezamizumab in a phase 1 study
Source: Orphanet J Rare Dis. 2022 Jul 9;17:259. doi: 10.1186/s13023-022-02405-7 (PMC9271258; doi:10.1186/s13023-022-02405-7)
Supplement: Supplementary file 2 — Additional file 2: Supplementary results. [file 13023_2022_2405_MOESM2_ESM.docx]

# Supplementary results. Description of outcomes in non-responders

## GGT assessments in patients with hepatic involvement

One patient with hepatic involvement and AL amyloidosis was a non-responder (119). GGT was stable throughout follow-up.

## eGFR assessments in patients with renal involvement

One patient with cardiac and renal involvement, and AL amyloidosis, was a non-responder (120). eGFR decreased to 57 mL/min/1.73 m^2^ at 7 months post dose.

## Cardiac assessments in patients with cardiac involvement

In the four non-responder patients with cardiac involvement (patients 120, 123, 124 and 125), NT-proBNP levels increased during or at the end of follow-up.

## Imaging assessments

In all three patients with ATTR (123, 124 and 125), DPD scans showed Grade 2 tracer uptake in the heart at the first visit recorded (post diagnosis/pre-baseline in patients 123 and 124, and 12.9 months post baseline in patient 125). At the second visit recorded, this remained at Grade 2 for patients 124 and 125 (11.0 and 27.6 months post baseline, respectively) but changed to Grade 1 in patient 123 (29.4 months post baseline).

In patient 119, amyloid load via SAP scan remained large throughout the study and follow-up period. In patient 120, overall amyloid load was moderate during the study and follow-up, although was recorded as ‘better’ at the last follow-up visit.
