# Supplementary material for: An observational, non-interventional study for the follow-up of patients with amyloidosis who received miridesap followed by dezamizumab in a phase 1 study
Source: Orphanet J Rare Dis. 2022 Jul 9;17:259. doi: 10.1186/s13023-022-02405-7 (PMC9271258; doi:10.1186/s13023-022-02405-7)
Supplement: Supplementary file 5 — Additional file 5: Table S3. 6MWD (m). [file 13023_2022_2405_MOESM5_ESM.docx]

# Table S3. 6MWD (m)

| **Patient number** | **Amyloid type** | **1^st^ value**  **post diagnosis** | **Value at baseline^*^** | **Value at 1^st^ follow-up** | **Value at 2^nd^ follow-up** | **Value at 3^rd^ follow-up** | **Value at 4^th^ follow-up** | **Value at 5^th^ follow-up** | **Value at 6^th^ follow-up** | **Value at 7^th^ follow-up** |
| --- | --- | --- | --- | --- | --- | --- | --- | --- | --- | --- |
| Sustained responders | | | | | | | | | | |
| 012 | AA | 385 | 460 | 481 | 446 | – | – | – | – | – |
| 107 | AL | 368^†^ | 368^†^ | 391 | 483 | 448 | – | – | – | – |
| 108 | AL | 506 | 552 | 583 | 583 | 600 | 575 | 514 | 626 | 644 |
| 110^‡^ | AL | 428 | 617 | 598 | 671 | 622 | 598 | – | – | – |
| 113^‡^ | AL | 477 | 489 | 531 | 529 | – | – | – | – | – |
| 116 | AL | 465 | 529 | 575 | 609 | 671 | 615 | 598 | – | – |
| 121 | AL | 391 | 508 | 466 | 520 | 563 | 520 | – | – | – |
| Declining responders | | | | | | | | | | |
| 102 | AFib | 506^†^ | 506^†^ | 536 | 506 | 545 | 544 | – | – | – |
| 104 | AFib | – | – | 500^§^ | 437 | 470 | – | – | – | – |
| 105 | AFib | 677 | 690 | 690 | – | – | – | – | – | – |
| 106 | AFib | 46 | 54 | 164 | 92 | – | – | – | – | – |
| 109 | ApoAI | 378 | 439 | 506 | 506 | 483 | 506 | 506 | 512 | 493 |
| 111^‡^ | AL | 516 | 467 | 391 | – | – | – | – | – | – |
| 114^‡^ | AL | 422 | 552 | 596 | 489 | 572 | 531 | – | – | – |
| 115 | AL | 224 | 333 | 350 | 391 | 304 | – | – | – | – |
| 117 | AFib | 564^†^ | 564^†^ | 500 | 492 | 593 | 470 | – | – | – |
| 118 | AL | 299 | 452 | 460 | 476 | 546 | 518 | 360 | – | – |
| Non-responders | | | | | | | | | | |
| 119 | AL | 508 | 588 | 552 | 612 | 621 | 593 | – | – | – |
| 120 | AL | 506 | 600 | 479 | – | – | – | – | – | – |
| 123 | ATTR  (hereditary) | 360 | 368 | – | – | – | – | – | – | – |
| 124 | ATTR  (wild-type) | 346 | 402 | 276 | 362 | 335 | 307 | – | – | – |
| 125 | ATTR  (wild-type) | 565^†^ | 565^†^ | 626 | 561 | 529 | 414 | 483 | 486 | – |
| Non-therapeutic dose in FIH study | | | | | | | | | | |
| 001 | AA | 483^†^ | 483^†^ | 501 | 489 | 483 | 552 | 578 | – | – |

Follow-up visits are presented in chronological order per parameter as given in the database. These visits will therefore occur at different times relative to baseline for each patient and parameter. Therefore, it is not possible to directly compare values at a particular visit between patients or between parameters within a patient.

^*^The latest value in the database pre-baseline. The baseline date was defined as the date of first pharmacologically active administration of dezamizumab (i.e. 200 mg in the session) in the FIH study. For patients who only received a non-pharmacologically active dose (i.e. <200 mg in the session), their baseline date was defined as the date of first administration of a non-pharmacologically active dose of dezamizumab.

^†^1^st^ value post diagnosis and value at baseline were measured at the same study visit.

^‡^Experienced clonal relapse during parent study and/or follow-up (see manuscript **Table 3** footnotes for details).

^§^A total of 3 6MWD values were recorded for this patient, but none were recorded as baseline data.

6MWD, 6-minute walking distance; AA, serum amyloid A; AFib, fibrinogen A alpha-chain; AL, immunoglobulin light chain;
ApoA1, apolipoprotein A-I; ATTR, transthyretin; FIH, first-in-human.
