# Supplementary material for: An observational, non-interventional study for the follow-up of patients with amyloidosis who received miridesap followed by dezamizumab in a phase 1 study
Source: Orphanet J Rare Dis. 2022 Jul 9;17:259. doi: 10.1186/s13023-022-02405-7 (PMC9271258; doi:10.1186/s13023-022-02405-7)
Supplement: Supplementary file 6 — Additional file 6: Table S4. Cardiac assessments (grey shading denotes subjects with cardiac involvement). [file 13023_2022_2405_MOESM6_ESM.docx]

# Table S4. Cardiac assessments (grey shading denotes subjects with cardiac involvement)

| **Patient number** | **Amyloid type** | **Cardiac involvement** | **Parameter** | **1^st^ value**  **post diagnosis** | **Value at baseline^*^** | **Value at 1^st^ follow-up** | **Value at 2^nd^ follow-up** | **Value at 3^rd^ follow-up** | **Value at 4^th^ follow-up** | **Value at 5^th^ follow-up** | **Value at 6^th^ follow-up** | **Value at 7^th^ follow-up** |
| --- | --- | --- | --- | --- | --- | --- | --- | --- | --- | --- | --- | --- |
| Sustained responders | | | | | | | | | | | | |
| 012 | AA | No | LV septum (mm) | 11 | 9 | 11 | 11 | – | – | – | – | – |
|  |  |  | LV posterior wall thickness (mm) | 11 | 9 | 10 | 10 | – | – | – | – | – |
|  |  |  | E:E’ | 7 | 9 | 10 | 8.4 | – | – | – | – | – |
|  |  |  | LVEF (%) | 75 | 65 | 70 | 70 | – | – | – | – | – |
| 107 | AL | No | LV septum (mm) | 10.7 | 9 | 8 | 9 | 10 | – | – | – | – |
|  |  |  | LV posterior wall thickness (mm) | 10.5 | 9 | 8 | 10 | 10 | – | – | – | – |
|  |  |  | E:E’ | – | 8 | 11.9 | 8 | 16 | – | – | – | – |
|  |  |  | LVEF (%) | – | 60 | 60 | 57 | 55 | – | – | – | – |
| 108 | AL | No | LV septum (mm) | 8 | 9 | 8 | 8 | 8 | 10 | – | – | – |
|  |  |  | LV posterior wall thickness (mm) | 9 | 9 | 9 | 8 | 8 | 10 | – | – | – |
|  |  |  | E:E’ | 6.8 | 6 | 6 | 7 | 5.481 | 6 | – | – | – |
|  |  |  | LVEF (%) | 61 | 58 | 66 | 60 | 60 | 60 | – | – | – |
| 110^†^ | AL | No | LV septum (mm) | 12 | 10 | 11 | 12 | 12 | – | – | – | – |
|  |  |  | LV posterior wall thickness (mm) | 11 | 11 | 11 | 8 | 12 | – | – | – | – |
|  |  |  | E:E’ | 6 | 6 | 6 | 6 | 9 | – | – | – | – |
|  |  |  | LVEF (%) | 58 | 67 | 60 | 60 | 60 | – | – | – | – |
| 113^†^ | AL | No | LV septum (mm) | 10 | 10 | 11 | – | – | – | – | – | – |
|  |  |  | LV posterior wall thickness (mm) | 11 | 10 | 10 | – | – | – | – | – | – |
|  |  |  | E:E’ | – | 12 | 10 | – | – | – | – | – | – |
|  |  |  | LVEF (%) | 57 | 60 | 60 | – | – | – | – | – | – |
| 116 | AL | No | LV septum (mm) | 9 | 10 | 8 | 9 | 9 | 10 | – | – | – |
|  |  |  | LV posterior wall thickness (mm) | 9 | 10 | 10 | 8 | 9 | 10 | – | – | – |
|  |  |  | E:E’ | 3.9 | 6 | 9.5 | 4.9 | 7.874 | 4.9 | – | – | – |
|  |  |  | LVEF (%) | 64 | 63 | 60 | 60 | 60 | 65 | – | – | – |
| 121 | AL | Yes | LV septum (mm) | 18 | 17 | 17 | 14 | – | – | – | – | – |
|  |  |  | LV posterior wall thickness (mm) | 18 | 18 | 17 | 14 | – | – | – | – | – |
|  |  |  | E:E’ | 17 | 18 | 31 | 17 | – | – | – | – | – |
|  |  |  | LVEF (%) | 59 | 53 | 48 | 45 | – | – | – | – | – |
| Declining responder | | | | | | | | | | | | |
| 102 | AFib | No | LV septum (mm) | 11 | 11 | 10 | 11 | 11 | – | – | – | – |
|  |  |  | LV posterior wall thickness (mm) | 11 | 10 | 10 | 10 | 11 | – | – | – | – |
|  |  |  | E:E’ | 7.5 | 11 | 10 | 7.6 | 13 | – | – | – | – |
|  |  |  | LVEF (%) | 67 | 60 | 59 | 65 | 55 | – | – | – | – |
| 104 | AFib | No | LV septum (mm) | 16 | 11 | 11 | 11 | – | – | – | – | – |
|  |  |  | LV posterior wall thickness (mm) | 11 | 10 | 11 | 11 | – | – | – | – | – |
|  |  |  | E:E’ | 6.3 | 5 | – | 6 | – | – | – | – | – |
|  |  |  | LVEF (%) | 63 | 60 | 60 | 60 | – | – | – | – | – |
| 105 | AFib | No | LV septum (mm) | 10 | 12 | 10 | – | – | – | – | – | – |
|  |  |  | LV posterior wall thickness (mm) | 11 | 10 | 10 | – | – | – | – | – | – |
|  |  |  | E:E’ | 7 | – | 10 | – | – | – | – | – | – |
|  |  |  | LVEF (%) | 60 | 60 | 60 | – | – | – | – | – | – |
| 106 | AFib | No | LV septum (mm) | 10 | 11 | 9 | 11 | – | – | – | – | – |
|  |  |  | LV posterior wall thickness (mm) | 11 | 10 | 10 | 11 | – | – | – | – | – |
|  |  |  | E:E’ | – | 7.6 | 10 | 13 | – | – | – | – | – |
|  |  |  | LVEF (%) | 57 | 52 | 57 | 55 | – | – | – | – | – |
| 109 | ApoAI | No | LV septum (mm) | 11 | 10 | 10 | 10 | 10 | 8 | 11 | – | – |
|  |  |  | LV posterior wall thickness (mm) | 11 | 10 | 10 | 10 | 9 | 9 | 9 | – | – |
|  |  |  | E:E’ | 6 | 7 | 6 | 7.6 | 7 | 5.3 | 8 | – | – |
|  |  |  | LVEF (%) | 77 | 63 | 68 | 60 | 66 | 63 | 55 | – | – |
| 111^†^ | AL | No | LV septum (mm) | 9 | 10 | 8 | 10 | – | – | – | – | – |
|  |  |  | LV posterior wall thickness (mm) | 10 | 10 | 8 | 10 | – | – | – | – | – |
|  |  |  | E:E’ | – | 11 | 10 | 11 | – | – | – | – | – |
|  |  |  | LVEF (%) | 70 | 69 | 55 | 65 | – | – | – | – | – |
| 114^†^ | AL | No | LV septum (mm) | 9 | 10 | 10 | 13 | 11 | 11 | – | – | – |
|  |  |  | LV posterior wall thickness (mm) | 9 | 9 | 9 | 13 | 10 | 10 | – | – | – |
|  |  |  | E:E’ | 5.4 | 6 | 4 | 4.9 | 6 | 6.745 | – | – | – |
|  |  |  | LVEF (%) | 74 | 63 | 60 | 64 | 66 | 60 | – | – | – |
| 115 | AL | No | LV septum (mm) | 12 | 12 | 12 | 12 | – | – | – | – | – |
|  |  |  | LV posterior wall thickness (mm) | 13 | 13 | 12 | 12 | – | – | – | – | – |
|  |  |  | E:E’ | 13 | 8 | 16 | 9.3 | – | – | – | – | – |
|  |  |  | LVEF (%) | 68 | 55 | 67 | 64 | – | – | – | – | – |
| 117 | AFib | No | LV septum (mm) | 12^‡^ | 12^‡^ | 12 | 13 | – | – | – | – | – |
|  |  |  | LV posterior wall thickness (mm) | 10^‡^ | 10^‡^ | 10 | 12 | – | – | – | – | – |
|  |  |  | E:E’ | 5.4^‡^ | 5.4^‡^ | 5 | 8.4 | – | – | – | – | – |
|  |  |  | LVEF (%) | 55^‡^ | 55^‡^ | 60 | 68 | – | – | – | – | – |
| 118 | AL | Yes | LV septum (mm) | 17 | 15 | 16 | 16 | 17 | 17 | 16 | 17 | – |
|  |  |  | LV posterior wall thickness (mm) | 16 | 15 | 16 | 17 | 17 | 16 | 16 | 17 | – |
|  |  |  | E:E’ | 22 | 24 | 20 | 20 | 20 | 16 | 22 | 18 | – |
|  |  |  | LVEF (%) | 45 | 52 | 50 | 62 | 50 | 49 | 50 | 38 | – |
| Non-responders | | | | | | | | | | | | |
| 119 | AL | No | LV septum (mm) | 11 | 12 | 12 | 10 | – | – | – | – | – |
|  |  |  | LV posterior wall thickness (mm) | 10 | 11 | 11 | 10 | – | – | – | – | – |
|  |  |  | E:E’ | 9.3 | 13 | 5 | 5 | – | – | – | – | – |
|  |  |  | LVEF (%) | 67 | 55 | 59 | 66 | – | – | – | – | – |
| 120 | AL | Yes | LV septum (mm) | 15 | 14 | 15 | – | – | – | – | – | – |
|  |  |  | LV posterior wall thickness (mm) | 15 | 14 | 15 | – | – | – | – | – | – |
|  |  |  | E:E’ | 15 | 20 | 26.9 | – | – | – | – | – | – |
|  |  |  | LVEF (%) | 57 | 61 | 60 | – | – | – | – | – | – |
| 123 | ATTR  (hereditary) | Yes | LV septum (mm) | 17 | 16 | 16 | 16 | 17 | 18 | 17 | 17 | – |
|  |  |  | LV posterior wall thickness (mm) | 16 | 17 | 17 | 17 | 17 | 17 | 18 | 18 | – |
|  |  |  | E:E’ | 22 | 20 | 25 | 19 | 15 | 21 | 21 | 21 | – |
|  |  |  | LVEF (%) | 45 | 39 | 39 | 41 | 48 | 34 | 35 | 53 | – |
| 124 | ATTR  (wild-type) | Yes | LV septum (mm) | 16 | 16 | 17 | 17 | 17 | 17 | 17 | 17 | – |
|  |  |  | LV posterior wall thickness (mm) | 16 | 16 | 17 | 17 | 18 | 17 | 17 | 17 | – |
|  |  |  | E:E’ | 11 | 13 | 14 | 11 | 16.8 | 9.3 | 13.3 | 11.9 | – |
|  |  |  | LVEF (%) | 47 | 48 | 60 | 50 | 39 | 46 | 45 | 45 | – |
| 125 | ATTR  (wild-type) | Yes | LV septum (mm) | 14^‡^ | 14^‡^ | 14 | 14 | 14 | 15 | 17 | 15 | 15 |
|  |  |  | LV posterior wall thickness (mm) | 13^‡^ | 13^‡^ | 14 | 14 | 14 | 15 | 15 | 15 | 15 |
|  |  |  | E:E’ | 6.6^‡^ | 6.6^‡^ | 9 | 8 | 10.871 | 11 | 13.1 | 8.61 | 11 |
|  |  |  | LVEF (%) | 60^‡^ | 60^‡^ | 55 | 60 | 55 | 49 | 49 | 60 | 60 |
| Non-therapeutic dose in FIH study | | | | | | | | | | | | |
| 001 | AA | No | LV septum (mm) | 11^‡^ | 11^‡^ | – | – | – | – | – | – | – |
|  |  |  | LV posterior wall thickness (mm) | 11^‡^ | 11^‡^ | – | – | – | – | – | – | – |
|  |  |  | E:E’ | 9.5^‡^ | 9.5^‡^ | – | – | – | – | – | – | – |
|  |  |  | LVEF (%) | 65^‡^ | 65^‡^ | – | – | – | – | – | – | – |

Follow-up visits are presented in chronological order per parameter as given in the database. These visits will therefore occur at different times relative to baseline for each patient and parameter. Therefore, it is not possible to directly compare values at a particular visit between patients or between parameters within a patient.

^*^The latest value in the database pre-baseline. The baseline date was defined as the date of first pharmacologically active administration of dezamizumab (i.e. 200 mg in the session) in the FIH study. For patients who only received a non-pharmacologically active dose (i.e. <200 mg in the session), their baseline date was defined as the date of first administration of a non-pharmacologically active dose of dezamizumab.

^†^Experienced clonal relapse during parent study and/or follow-up (see manuscript **Table 3** footnotes for details).

^‡^1^st^ value post diagnosis and value at baseline were measured at the same study visit.

AA, serum amyloid A; AFib, fibrinogen A alpha-chain; AL, immunoglobulin light chain; ApoA1, apolipoprotein A-I; ATTR, transthyretin; E:E’, early mitral inflow velocity and mitral annular early diastolic ratio; FIH, first-in-human; LV, left ventricle; LVEF, left ventricular ejection fraction.
