# Supplementary material for: An observational, non-interventional study for the follow-up of patients with amyloidosis who received miridesap followed by dezamizumab in a phase 1 study
Source: Orphanet J Rare Dis. 2022 Jul 9;17:259. doi: 10.1186/s13023-022-02405-7 (PMC9271258; doi:10.1186/s13023-022-02405-7)
Supplement: Supplementary file 7 — Additional file 7: Table S5. Involved free light chains (iFLC) in mg/L at follow-up visits in patients with AL amyloid. [file 13023_2022_2405_MOESM7_ESM.docx]

# Table S5. Involved free light chains (iFLC) in mg/L at follow-up visits in patients with AL amyloid

| **Patient number** | **FLC** | **1^st^ value**  **post diagnosis** | **Value at baseline^*^** | **Value at 1^st^ follow-up** | **Value at 2^nd^ follow-up** | **Value at 3^rd^ follow-up** | **Value at 4^th^ follow-up** | **Value at 5^th^ follow-up** | **Value at 6^th^ follow-up** | **Value at 7^th^ follow-up** | **Value at 8^th^ follow-up** | **Value at 9^th^ follow-up** |
| --- | --- | --- | --- | --- | --- | --- | --- | --- | --- | --- | --- | --- |
| Sustained responder | | | | | | | | | | | | |
| 107 | Kappa | 22.1 | 20.7 | 22.2 | 24.3 | 24.5 | 27.6 | – | – | – | – | – |
| 108 | Kappa | 40.7 | 313.5 | 12 | 11.3 | 14 | 16.3 | 16.4 | 14.7 | 15.3 | – | – |
| 110^†^ | Lambda | 495 | 34 | 15.8 | 22.7 | 33.9 | 40.3 | 36.5 | 244.4 | 29.8 | – | – |
| 113^†^ | Kappa | 131 | 40.5 | 83.7 | 28.3 | 15.6 | – | – | – | – | – | – |
| 116 | Lambda | 43.4 | 36.5 | 34.2 | 45.6 | 37 | 36.3 | 34.9 | 38.3 | 36.1 | 32.6 | – |
| 121 | Lambda | 431 | 23.6 | 23.8 | 25.3 | 23 | 9.3 | 18.6 | 17.5 | – | – | – |
| Declining responder | | | | | | | | | | | | |
| 111^†^ | Lambda | 17.1 | 12.3 | 16.3 | 22 | 25.2 | 24.7 | 31.7 | – | – | – | – |
| 114^†^ | Kappa | 27 | 39.4 | 52.4 | 44 | 50 | 59.6 | 65.8 | 60.7 | 68 | – | – |
| 115 | Lambda | 9.1 | 18.4 | 31 | 26.9 | 23.5 | 29 | 25 | – | – | – | – |
| 118 | Lambda | 10.6 | 6.1 | 26.5 | 35.1 | 35.1 | 42.4 | 45.6 | 72.4 | 76.7 | 57.2 | 52.2 |
| Non-responder | | | | | | | | | | | | |
| 119 | Kappa | 9.2 | 25.3 | 27.3 | 26.7 | 22.3 | 28.3 | 26.5 | – | – | – | – |
| 120 | Lambda | 31.2 | 50.8 | 76.6 | 48.6 | – | – | – | – | – | – | – |

K, kappa; L, lambda; iFLC, involved free light chain.

Follow-up visits are presented in chronological order per parameter as given in the database. These visits will therefore occur at different times relative to baseline for each patient and parameter. Therefore, it is not possible to directly compare values at a particular visit between patients or between parameters within a patient.

^*^The latest value in the database pre-baseline. The baseline date was defined as the date of first pharmacologically active administration of dezamizumab (i.e. 200 mg in the session) in the FIH study. For patients who only received a non-pharmacologically-active dose (i.e. <200 mg in the session), their baseline date was defined as the date of first administration of a non-pharmacologically active dose of dezamizumab.
^†^Experienced clonal relapse during parent study and/or follow-up (see manuscript **Table 3** footnotes for details).
